# Effects of whole-body vibration on proxies of muscle strength in old adults: a systematic review and meta-analysis on the role of physical capacity level

**DOI:** 10.1186/s11556-015-0158-3

**Published:** 2015-12-08

**Authors:** Slavko Rogan, Eling D. de Bruin, Lorenz Radlinger, Christine Joehr, Christa Wyss, Neil-Jerome Stuck, Yvonne Bruelhart, Rob A. de Bie, Roger Hilfiker

**Affiliations:** Bern University of Applied Sciences, Discipline Physiotherapy, Bern, Switzerland; Department of Epidemiology, CAPHRI School for Public Health and Primary Care, Maastricht University, PO Box 616, 6200 MD, Maastricht, The Netherlands; Centre for Evidence Based Physiotherapy, Maastricht University, PO Box 616, 6200 MD, Maastricht, The Netherlands; Department of Health Sciences and Technology, Institute of Human Movement Sciences and Sport, ETH, Zurich, Switzerland; School of Health Sciences, University of Applied Sciences and Arts Western, Switzerland, Valais

**Keywords:** WBV, Isometric maximum voluntary contraction, Dynamic maximum voluntary contraction, Power, Rate of force development, Functional strength

## Abstract

**Background:**

Dynapenia (age-associated loss of muscle strength not caused by neurologic or muscular diseases) and functional limitations (e.g. climbing stairs, chair rising) are important problems in elderly persons. Whole body vibration, used as an adjunct to classical resistance training or even as a stand-alone alternative, might help to reduce these problems. Its value might be highest in elderly persons with very low function, where whole body vibration can be used as a skilling up training until more conventional exercise types are possible. This systematic review and meta-analysis summarized the current evidence for whole-body vibration interventions on isometric maximum voluntary contraction, dynamic strength, power, rate of force development and functional strength in elderly categorised in different subgroups based on function levels.

**Methods:**

An extensive literature search was carried out in February 2014 and repeated in February 2015 at PubMed, Cochrane Central Register of Controlled Trials, Physiotherapy Evidence Database and CINAHL electronic databases. The International Clinical Trials Registry Platform from the World Health Organization was also searched. Randomized controlled trials measuring isometric maximum voluntary contraction, dynamic strength, power, rate of force development and functional strength in studies using WBV intervention in 65 years or older elderly individuals were included. The methodological quality of included studies was assessed using the Cochrane Collaboration’s tool for assessing Risk of Bias. Studies were classified based on the level of physical capacitiy of the participants as “Go-Go”, “Slow-Go” or “No-Go”. Data were pooled using a random effects model.

**Results:**

Thirty-eigth articles of moderate methodological quality were included. The vibration modes for sinusoidal vertical whole-body vibration was between 25 and 40 Hz, the amplitude varied from 2 to 4 mm. Sinusoidal side-alternating -whole-body vibration revealed frequencies from 2.5 to 35 Hz with amplitudes ranging from 0.05 to 12 mm. Stochastic resonance whole-body vibration used frequencies between 3 and 6 Hz. Effect sizes in Go-Go were moderate after vertical sinusoidal Whole-body vibration compared to non-training control groups for isometric maximum voluntary contraction with effect size 0.48 (95 % CI 0.33 to 0.63) and for Dynamic Strength with effect size 0.47 (95 % CI 0.06 to 0.88). Side-alternating sinusoidal whole body vibration showed moderate effect sizes with 0.69 (95 % CI 0.32 to 1.06) for isometric maximum voluntary contraction, 0.50 (95 % CI 0.07 to 0.92) for power, 0.40 (95 % CI 0.16 to 0.64) for Rate of Force Development and 0.42 (95 % CI 0.13 to 0.71) for Functional Strength compared to non-exercise control. The analysis for Slow-Go showed for stochastic resonance whole-body vibration and Functional Strength an effect size of 0.97 (95 % CI −0.07 to 2.00) compared to non-exercise control in one study. No-Go showed for stochastic resonance whole-body vibration a moderate effect size with 0.50 (95 % CI −0.32 to 1.33) for Functional Strength compared to non-exercise control.

**Conclusions:**

Whole-body vibration shows beneficial effects, mainly in the No-Go group elderly compared to non-training control and conventional strength training groups. The results suggest that WBV can be used as a skilling-up exercise in participants not able to perform standard exercises. Further studies with the various types of WBV in various sub-populations of elderly persons are needed to determine the most effective vibration modes.

**Trial registration:**

Registration number: CRD42013006489.

**Electronic supplementary material:**

The online version of this article (doi:10.1186/s11556-015-0158-3) contains supplementary material, which is available to authorized users.

## Background

Aging is associated with a decrease of muscle strength and power [1–3]. The term dynapenia, coined by Manini and Clark [3–5], best describes the condition of decreased muscle strength and power instead of the term sarcopenia. The latter only refers to an age-related loss in skeletal muscle mass. Muscle weakness is related to falls, lower walking speed, functional limitation, a decrease in mobility, and disability [6–8]. In this context, the elderly are viewed as a group of people in need [9]. When physical functioning is concerned there often is a mismatch between chronological age and biological age. Chronological age is not necesarilly related to physical capabilities. For this reason, a classification of elderly based on physical abilities; e.g. physical and mental functions is more appropriate.

Zeyfang and Braun [10] classified older adults as “*being an independent person*” (Go-Go); “*being a needy person with a slight handicap*” (Slow-Go); and “*being a person in need of care with severe functional limitation*” (No-Go). The need for care may be defined as depending permanently on assistance (No-Go) or depending on support in everyday activities such as dressing, body care, eating, using the toilet, mobility, and planning the day (Slow-Go) [11].

The ability of elderly individuals to perform basic activities of daily life is crucial for their ability to exist independently [12]. To improve and/or enable performance of basic activities of daily life, exercise programs are indicated. Sensorimotor training and resistance exercises are effective methods to increase muscle mass and strength in the elderly [13]. Whole-body vibration (WBV) can be used as a sensorimotor training regimen. The impact of WBV on the body is low according to indicators such as blood pressure, heart rate, lactate, and O_2_ uptake [14–16]. Systematic reviews concluded that, compared to more demanding interventions, WBV might be a safer and less fatiguing type of exercise [17] with a beneficial effect on movement skills [18].

Three types of WBV are used based on the amount of vibrating plates [18, 19]. Sinusoidal vertical whole-body vibration (SV-WBV) and sinusoidal side-alternating whole-body vibration (SS-WBV) use a single vibrating platform, whereas stochastic resonance whole-body vibration (SR-WBV) expects the trainees to stand on two platforms. During sinusoidal WBV the participants stand on a platform that vibrates vertically (SV-WBV) or to the side alternating (SS-WBV) with a high frequency between 20 and 50 Hz and an amplitude between 2 and 14 mm [20]. SR-WBV vibrates with frequencies between 1 and 12 Hz and an amplitude between 3 and 6 mm while the feet of the participants are placed on two independent powered and stochastic vibrating platforms [20].

In recent years, WBV has been introduced as a training method to improve muscle power and strength [21–23]. Several systematic reviews [24, 25] report on strength-related outcomes. However, no review has considered muscle strength related outcomes in a comparison of WBV against non-exercising control or conventional exercise groups and no review evaluated the effects separately for groups differing in initial levels of physical functioning, e.g. the three groups “Go-Go, Slow-Go and No-Go”. This is of relevance, however, since training principles would let us expect that those with the lowest level of fitness have greatest room for improvement. In other words, improvement in the outcome of interest will be greatest in those with lower initial values [26]. Furthermore, no review includes stochastic resonance WBV.

For clinicians, a systematic overview about the relevance and indication for application of SV-WBV, SS-WBV or SR-WBV and how it might be applied for Go-Go, Slow-Go and No-Go elderly individuals is lacking. Therefore, the aims of this systematic review are to provide 1) an overview of the current studies on WBV, 2) to determine the effects of WBV on strength or power in Go-Go, Slow-Go and No-Go elderly individuals and 3) give recommendations on available evidence for practical use. We hypothesized that WBV differently effects on measures of strength and power in Go-Go, Slow-Go and No-Go.

## Methods

### Data sources and searches

Inclusion criteria and analysis methods were developed and documented in a protocol prior to the current review. Included were elderly over 65 years of age; excluded were Geriatric diseases (Parkinson disease, Stroke, Multiple sclerosis), studies applying electrical current vibration or vibration with shoe insoles. More detailed information on the protocol including a link to the search strategy can be found on http://www.crd.york.ac.uk/PROSPERO/display_record.asp?ID=CRD42013006489 (PROSPERO registration number 2013:CRD42013006489). This systematic review and meta-analysis followed the PRISMA guidelines [27].

A first literature search of electronic databases was repeatedly carried out from January 2013 to February 2014 in the PubMed, CENTRAL (Cochrane Central Register of Controlled Trials), Physiotherapy Evidence Database (PEDro) and CINAHL electronic databases. The International Clinical Trials Registry Platform from the World Health Organization (WHO) was also searched. In February 2015 the search was repeated shortly before submission of the manuscript to ensure inclusion of most recent relevant material in the review. Additionally, a manual search of the reference lists of retrieved publications was conducted. English and German language restrictions were imposed upon the search.

### Systematic search

The following keywords and combinations according to the PICO-model [28] were used in the search strategy:

- Population: elderly, aged, dwelling home, nursing home, human research.
- Intervention: Whole Body Vibration, WBV, noise, random vibration, RCT.
- Comparator: WBV against control intervention (non-exercise or exercise on a level too low to effect on muscle [29]), WBV against conventional strength training intervention.
- Outcome: strength, maximal voluntary contraction, power, rate of force development, performance, falls. Our search terms are detailed in Additional file 1.

Based on the four PICO components, a final question was stated as: For an older adult with diminishing physical capacity, will whole body vibration (WBV) exercise as compared to non- or conventionally exercising older adults improve muscle strength and/or power?

The following aspects were operationalized: 1) assessment of the quality and internal validity of the studies reviewed; 2) description of the assessments used to document the effect of WBV on isometric maximal voluntary contraction (IMVC), dynamic strength (DS), power, rate of force development (RFD) and functional strength (FS); 3) composition of the WBV training parameters; and 4) conclusion about clinical relevance in general.

### Study selection

Five independent reviewers (CJ, CW, NJS, SR, RH) screened the titles and abstracts for eligibility. They screened for randomized controlled trials (RCTs) measuring maximal voluntary contraction, power and rate of force development in studies using WBV intervention in elderly individuals (mean age at least 65 years). Full text articles in English or German were eligible for inclusion. Healthy elderly participants and all clinical outcome measures of IMVC, DS, power, RFD, and FS were included in this review. Studies describing vibrations applied by electrical current or vibrating insoles, and patient series were excluded.

In the event of missing data, additional information was requested from the corresponding authors in order to include these data in our meta-analysis.

### Data extraction

In addition, general characteristics of the studies were extracted. Five authors (CJ, CW, NJS, SR, RH) independently abstracted the following information from each of the studies included in this review: 1) design and sample; 2) inclusion criteria; 3) training parameters (i. e. duration, frequency, intensity of WBV); 4) type of vibration plate; 5) change in strength, power, RFD; 6) conclusions of the studies and statistical significance.

### Methodological quality assessment of studies

The methodological quality of the included articles was rated with the “Cochrane Collaboration tool for assessing risk of bias” (RoB) [30] to assess the risk of over- or under-estimating the effects of an intervention [31].

Nine items, with each having three rating categories, were scored and divided into six domains of bias (Fig. 2): (1) low ROB, (2) unclear ROB and (3) high ROB. Rating (1) is unlikely to alter the results significantly, (2) raises some doubt about the results and (3) seriously weakens confidence in the results. With insufficient information on an item, the score given was “high risk”. The arbitration of a third reviewer was used in the event of any disagreement between the reviewers (YB, RH) for both ratings.

### Data synthesis and analysis

Most outcomes of interest were presented as continuous data (mean values and SD or mean changes). For the meta-analysis of the present study the standardized mean difference (SMD) and 95 % confidence interval (CIs) of the post-intervention values or changes in scores were used for all comparisons. SMDs were pooled with a random effects model. The magnitude of the effect sizes for the between groups comparisons, calculated by SMDs are interpreted as follows: an effect size (d) around 0.2 indicates a small effect size, around 0.5 a medium effect size, and around 0.8 a large effect size [32]. If only one study was identified or data were not presented in a format that allowed inclusion in the dataset, results of individual studies are presented. If studies reported more than one IMVC, DS, power or RFD, and FS; then we only extracted the first outcome data on a hierarchy of outcomes:

- IMVC: 1. isometric knee extension, 2. isometric hip extension, 3. isometric leg press; DS: 1. dynamic leg press, 2. dynamic knee extension (lowest speed), 3. dynamic hip extension; Power: 1. leg press, 2. knee extension; RFD: 1. counter movement jump, 2. squat jump, 3. leg press,; FS: 1. chair rise time, 2. chair rise repetition, 3. chair rise power, 4. stair climb, 5. wall squat. Subgroup analyses were undertaken to assess the effects of WBV on IMVC, DS, power, RFD and FS in Go-Go, Slow-Go and No-Go.

Heterogeneity was assessed by forest plots and the I^2^ statistics. Values >25 % indicate small, >50 % middle and >75 % considerable heterogeneity [33]. All other information was summarized and analysed qualitatively. Stata (version 13) was used for all meta-analyses.

## Results

### Study selection

**Fig. 1:**
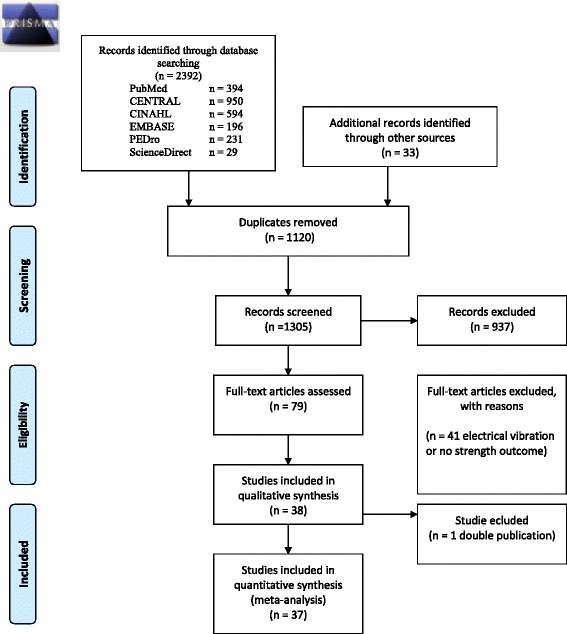
Flow diagram

The literature search yielded 1383 studies (PubMed *n* = 394, Central *n* = 163, Cinahl = 446, Embase = 174, Pedro *n* = 144, ScienceDirect *n* = 29, WHO International Clinical Trials Registry Platform *n* = 33). After identification of 367 duplicates, 1016 titles and abstracts were screened. Seventy-nine studies remained for further full-text analysis. Subsequently, 41 studies were excluded because they did not address strength, power or rate of force development or included participants with diseases. Finally, 38 full text papers [20, 23, 34–64] were included for this review and 37 were used for the meta-analysis (Fig. 1).

### Study characteristics

The major characteristics of the included studies are summarized in Table 1. Three papers investigated the effects on force by vertical and side-alternating sinusoidal WBV [35, 46, 66]. Five studies included more than 100 participants. Leung et al. [66] (*n* = 596), Boegarts et al. [38] (*n* = 180), Kemmler et al. [44] (*n* = 151) von Stengel [59] (*n* = 151) and Sitjà-Rabert et al. [64] (*n* = 117). The other included studies had a small sample size of less than 100 participants. The used strength outcomes varied across all included studies.

**Table 1:** Study characteristics of the included studies

Study	Participants (N, sex distribution); mean age (± SD)	Protocol exercise	Outcome measures	Within GE	Between GE
				(yes/no)	(yes/no)
Vertical sinusoidal vibration					
Amaral et al. [78]	WBV: 9 ♀; 76.6 (±11.8)	WBV: isometric squat	FS: CR over 30 s	No	No
	Con: 9 ♀; 78.6 (±10.4)	Con: daily activities routines			
Álvarez- Barbosa et al. [62]	WBV: 15, 12 ♀; 84.0 (±3.0)	WBV: dynamic exercise lunge, squat, calf raises, left and right pivot in a front and lateral position, step up and down.	FS: CR over 30 s	Yes	Yes
	Con: 15, 11 ♀; 86.0 (±7.5)	Con: no change lifestyle			
Bautmans et al. [34]	WBV: 10, 8 ♀; 76.6 (±11.8)	WBV: static position exercises (lunge squats, squats, deep squats, wide stance squats, calves, calves deep) druing WBV	DS: (N)	Yes	NO
			Power: (W), work (J)		
	Sham: 11, 6 ♀; 78.6 (±10.4)		RFD: (N/s) at 40 and 60 cm/s		
Beck et al. [35]	WBV: 15 ♀; 68.5 (±8.6)	Pos: full extension	FS: wall squat with dominant leg and non dominant leg & CR over five repetitions (s)	Yes	No
	Con: 15 ♀; 74.2 (±8.1)	Con: no vibration			
Bogaerts et al. [37]	WBV: 25 ♂; 66.9 (±0.7)	WBV: squat, deep squat, wide stance squat, toesstand, toes-stand deep, one-legged squat, and lunge.	IMVC: knee extension (Nm)	Yes	Yes
	Ex: 25 ♂; 67.4 (±0.9)				
	Con: 32 ♂; 68.6 (±1.0)	Ex: cardio exercise, strength and balance training, flexibility exercise	RFD: CMJ (cm) on a contact mat.		
		Con: no change lifestyle			
Bogaerts et al. [38]	WBV: 70; 66.8	WBV: exercises for upper and lower body	IMVC: knee extension (Nm)	Yes	No
	Ex: 49: 66.8	Ex: cardio exercise, strength and balance training, flexibility exercise			
	Con: 61; 67.8 (ratio ♂:♀ = 1.5:1 for the total sample)	Con: no change life style			
Boegarts [36]	WBV 1: 26 ♀; 80.3 (±5.3)	WBV: squat, deep squat, wide stance squat, toes stand and one legged squat	Physiological Profile Assessment [76]:	Yes	No
	WBV 2: 28 ♀; 79.8 (±5.3)	Con 1 & 2: no change life style	IMVC: knee extension (kg)		
	Con 1: 29 ♀; 78.7 (±5.6)				
	Con 2: 28 ♀; 79.6 (±5.2)				
Corrie et al. [65]	WBV: 21, 13 ♀; 81.9 (±5.7)	WBV: standing position, with bent knees and Otago Exercise programm	Power: (W/kg body weight) leg press	Yes (power & CR)	Yes (power)
	Sham: 20, 16 ♀; 79.1 (±7.8)	Sham: Otago Exercise program which consisted of 6 weekly visits	RFD: CMJ (N/kg body weight		
			FS: CR over five repetitions		
Gomez-Cabello [42]	WBV: 24	WBV: squat position	Senior Fitness Test battery and Eurofit Testing Battery [77]:	Yes	No
	Con: 25 (20 ♂, 29 ♀)	Con: no change life style	FS: CR over 30 s (repetition)		
Kemmler et al. [44]	WBV: 50 ♀; 68.8 (±3.6)	WBV: static and dynamic exercise (toe stand, squat)	IMVC: leg press (N)	Yes	Yes
	Ex: 50 ♀; 68.6 (±3.0)	Ex: static and dynamic exercise (toe stand, squat) without vibration	Power: leg press (W/kg)		
	Con: 51 ♀; 68.1 (±2.7)	Con: exercise and relaxation program once a week (30× 60 min)	RFD: leg press (N/ms) & Squat jump (jump height, cm)		
Kennis et al. [45]	WBV: 23 ♂	WBV: static and dynamic squat, deep squat, wide stance squat, 1-legged squat, lunge, toes-stand, toes-stand deep, moving heels.	IMVC: at 120° knee extension (Nm)	Yes	No
	Ex: 20 ♂				
	Con: 29 ♂	Ex: 60–90 min aerobic, resistance, balance, and flexibility exercises	DS: concentric dynamic knee extension (Nm) at a movement velocity at 120°/s.		
		Con: no change life style	RFD: counter movement jump (high, cm)		
Klarner et al. [46]	WBV: 36 ♀; 68.1 (±4.0)	WBV: dynamic exercises	IMVC: with leg press (N)	Yes	Yes
	Con: 36 ♀; 67.6 (±4.13)	Con: 1/w low gymnastic exercise & relaxation exercise	RFD: with Counter movement jump (CMJ, jump height, cm)		
Lachance [47]	WBV: 26; 70.4 (±7.7)	WBV: static squats (60°), lunges (60°) and heel raises.	FS: CR over 30 s	Yes	No
	Ex: 29; 75.9 (±7.2)	Ex: static squats (60°), lunges (60°) heel raises, bicep curls, tricep extensions,			
	(33 ♂, 22 ♀)	Exercises were progressive in nature by safely increasing the number of repetitions completed and/or weight of the dumbbells.			
Leung et al. [66]	WBV: 280 ♀; 74.2 (±7.0)	WBV: standing upright without knee banding	IMVC: knee extensor (kg)	Yes	Yes
	Con: 316 ♀; 71.0 (±7.0)	Non: no change life style			
Machado et al. [48]	WBV: 13 ♀; 79.3 (±7.3)	WBV: static and dynamic exercise (half-squat (120–130°), deep squat (knee angle 90°), a wide-stance squat and calves.	IMVC: leg extensor (N)	Yes	Yes
	Con: 13 ♀; 76.2 (±8.4)	Con: no change life style	Power: output at three relatives loads: 20, 40, 60 % of the IMVC.		
Mikhael et al. [49]	WBV1: 6, 4 ♀; 63.3 (±7.6)	WBV: WBV1 with flexed knees at 20° and WBV 2 with extended knees.	DS: one repetition maximum (1RM) leg press (N), relative strength (kg/kg), leg press strength (kg)	Yes	Yes
	WBV2: 5, 3 ♀; 69.0 (±7.6)	Sham: flexed knees at 20° without vibration	Power: (W) and velocity (cm/s) were measured at 20, 30, 40, 50, 60, 70, 80, 90, and 100 % of current 1RM.		
	Sham: 8, 4 ♀; 62.3 (±8.8)		FS : CR over 30 s		
Roelants et al. [53]	WBV: 30 ♀; 64.6 (±0.7)	WBV: high squat (120° and 130°, deep squat (90°), wide-stance squat and lunge.	IMVC: (0°/s) torque (Nm) of knee extensor	Yes	No
	Ex: 30 ♀; 63.9 (±0.8)	Ex: resistance exercise	DS: dynamic extension-flexion movements (torque: N/m) between 90 and 160° at a velocity of 50, 100 and 150°/s.		
	Con: 29 ♀; 64.2 (±0.6)	Con: no change life style	RFD: jump height (mm) on a contact mat		
Sitjà-Rabert et al. [64]	WBV: 59; 64.6 (±0.7)	WBV: static/dynamic exercises.	FS: CR over five repetitions	Yes	No
	Ex: 58; 63.9 (±0.8)	Ex: static and dynamic exercise			
	(Total sample 67 % ♀)				
Verschueren et al. [58]	WBV: 25 ♀; 64.6 (±3.3)	WBV: static and dynamic knee-extensor exercises like squat, deep squat, wide-stance squat, one-legged squat and lunge.	IMVC: knee-extension	Yes	Yes
	Ex: 22 ♀; 63.9 (±3.8)	Ex: warm-up, resistance training knee-extensor on a leg extension and a leg press machine. Designed to the guideline of the American College of Sports Medicine	DS: isokinetic extension-flexion movements for maximal DS (peak torque N/m) at a velocity of 100°/s between of 90 and 160° joint angle.		
	Con: 24 ♀; 64.2 (±3.1)	Con: no change life style			
Verschueren et al. [57]	WBV: 28 ♀; 79.8 (±5.3)	WBV: static and dynamic knee-extensor exercises like squat, deep squat, wide-stance squat, one-legged squat and toe-stance.	IMVC: Knee-extension (Nm)	Yes	No
	Con: 28 ♀; 79.6 (±5.2)	Con: no change in life style	DS: Knee-extension (Nm).		
Sidealternating sinusoidal vibration					
Beck et al. [35]	WBV: 17 ♀; 68.9 (±70)	Pos: static with slightly bended knees	FS: wall squat dominant leg (DL) and non dominant leg (NDL) & CR over five repetitions (s)	Yes =	No
	Con: 15 ♀; 74.2 (±8.1)	Con: no vibration			
Calder et al. [39]	N: 41, 30 ♀; 80.1	WBV: stand with slightly bended knees (35° flexion) & Physiotherapy	FS: CR	Yes	No
		Con: Physiotherapy			
Corrie et al. [65]	WBV: 21, 16 ♀; 81.9 (±5.7)	WBV: standing position, with bent knees and Otago Exercise	Power: (W/kg body weight) leg press	No	No
	Sham: 20, 8 ♀; 79.1 (±7.8)	Sham: Otago Exercise program which consisted of 6 weekly visits	RFD: CMJ (N/kg body weight		
			FS: CR over five repetitions		
Furness and Maschette [40]	WBV1: 18 (1/week)	WBV: static with 70 knee flexion	FS: CR	Yes (for WBV2 and WBV3)	No
	WBV2: 18 (2/week)	Con: no vibration			
	WBV3: 19 (3/week)				
	Age: 72 (±8)				
	Con: 18 (0/wk)				
	(Total sample 38 ♂, 35 ♀)				
Furness et al. [41]	WBV: 19	WBV: static with 70° Kneeflexion	FS: CR	Yes	No
	Con: 18	Con: no exercise			
	(Total sample 16 ♂, 21 ♀)				
Iwamoto et al. [43]	WBV: 26 ♀; 72.4 (±8.1)	WBV: stands with bended knee and hips	FS: CR over 5 times	Yes	No
	Con: 26 ♀; 76.0 (±7.4)	Con. No exercise			
Klarner et al. [40]	WBV: 36 ♀; 67.9 (±3.78)	WBV: dynamic exercises	IMVC: Hip & Knee extension (N)	Yes	Yes
	Con: 36 ♀; 67.6 (±4.13)	Con: 1/w low gymnastic exercise & relaxation exercise	RFD: CMJ (jump height, cm)		
Ochi et al. [67]	WBV: 10 ♀; 80.9 (±2.8)	WBV: dynamic exercises	IMVC: Quadiceps muscle dominant leg	Yes	No
	Ex: 10 ♀; 80.2 (±3.3)	Con: dynamic exercise: half squat, heel rise, toe up.			
Raimundo et al. [50]	WBV: 14 ♀; 66 (±6)	WBV: static with knee angle 120°	DS: dynamic maximal unilateral strength at 60 and 300 °/s for concentric and eccentric at 60 °/s (Peak torque (Nm/kg).	Yes	Yes
	Ex: 13 ♀; 66 (±4)	Ex: walk-based-programme	Power (W).		
		Walking over 60 m with two sets with 70–75 % of their maximal heart rate.	RFD: mixed counter movement jump on		
			Ergo Jump Platform (Bosco System, Italy)		
			FS: CR over three repetitions.		
Rees et al. [51]	WBV: 15; 74.3 (±5.0)	WBV: static squats over 4 weeks, than dynamic squats and calf raises over 4 weeks.	DS: angular velocity 60°/s for knee and hip and the angle joint was tested at 30 °/s.	Yes	Yes
	Ex: 13; 73.1 (±4.1)	Ex: static squats over 4 weeks, than dynamic squats and calf raises over 4 weeks, without vibration.	FS: CR over five repetition		
	Con: 15; 73.1 (±4.6)				
	(Total sample 23 ♂, 20 ♀)				
Rees et al. [52]	WBV: 15; 74.3 (±5.0)	Con: only walking WBV: static and dynamic exercise (squats, calf raises)	DS: as torque (Nm/kg)	Yes	No
	Ex: 13; 73.1 (±4.1)	Ex: same exercise without vibration	maximum isokinetic power (W/kg) angular velocity for the hip and knee was 60°/s, with the ankle joint tested at 30°/s.		
	(No sex distribution information)				
Russo et al. [55]	WBV: 17 ♀	WBV: static, knees slightly flexed	DS: strength (N), acceleration of the centre of gravity (COG) was calculated as the ratio of force (N) and body mass (kg).	Yes	No
	Age: 60.7 (±6.1)	Con: no change in life style	RFD: starting from a standstill, jumped as high as possible and landed (W).		
	Con: 16 ♀				
	Age: 61.4 (±7.3)				
Sievänen et al. [61]	WBV: 8, 7 ♀	WBV: dynamic exercise such as slight squatting, toe raises, lateral weigth transfer.	FS: SPPB	Yes	NO
	Age: 84.4 (±6.3)	Ex: light squatting, toe raises or weight transfer forward and lateral weight transfer on WBV.			
	Sham: 7, 5 ♀				
	Age: 83.6 (±8.9)				
Stolzenberg et al. [56]	WBV: 30 ♀; 67.5 (±3.8)	WBV: static standing with slightly bent knees and hips, continuous squatting from erect standing to 90° knee flexion or static stance in 90° knee flexion	Power: CMJ (W/kg)	Yes	No
	Con: 30 ♀; 65.5 (±4.3)	Con: balance exercise like Romberg, tandem and single-leg stance.	RFD: CMJ jump height (cm)		
			FS: 1-leg hopping and CR over five repetitions		
von Stengel et al. [59]	WBV: 50♀; 68.8 (±3.6)	WBV: heel rise, one-legged deep squat, and leg abduction	MVC: leg press (N)	Yes	Yes
	Ex: 50 ♀; 68.6 (±3.0)	Ex: heel rise, one-legged deep squat, and leg abduction without vibration	RFD: CMJ (W/Kg)		
	Con: 51 ♀; 68.1 (±2.7)	Con: exercise and relaxation program once a week in blocks of 10 weeks with breaks			
Zhang et al. [60]	WBV: 19, 2 ♀; 85.8 (±3.6)	WBV: different to their function. Who could stand: partial squat position with slight hip, knee and ankle joint flexion. Who could not stand independently, same position, but were allowed to hold the support bar with their hands.	IMVC: M. quadriceps	Yes	Yes
	Con: 18, 3 ♀; 84.7 (±3.7)	Ex: usual care, physical therapy (ultrasound therapy, electrical stimulation, etc.) and routine exercises, such as pedalling training with regular dosage and time of treatments.	FS: CR over 30 s		
Stochastic resonance vibration					
Kessler et al. [23]	WBV: 10, 8 ♀; 77 (±7.7)	static (e.g. normal stance, semi-tandem, one leg stance) and dynamic standing (e.g. squat)	IMVC: knee-extension (N)	Yes	Yes
	Sham:10, 8 ♀; 81 (±5.7)		RFD: knee-extension (N/s)		
			FS: SPPB		
Rogan et al. [54]	WBV: 10; 77 (±7.7)	static standing with slightly bent knees and hips	FS: CR (1 time)	Yes	No
	Sham:10; 81 (±5.7)				
	(No sex distribution information)				
Rogan et al. [20]	WBV: 5; 77 (±7.7)	static standing with slightly bent knees and hips	IMVC: knee-extension (N)	Yes	No
	Sham: 4; 81 (±5.7)		RFD: knee-extensor (N/s)		
	(Total sample 4 ♂, 5 ♀)		FS: SPPB		

*Abbreviation*: *Con* control group, *EX* exercise group, *Sham* sham group; *SD* standard deviation, *GE* group effests, *mo* month, *wk* week, *WBV* whole-body vibration, *Pos* position, *s* seconds, *IMVC* isometric maximal voluntary contraction, *DS* dynamic maximal strength, *RFD* rate of force development, *FS* functional strength, *CR* chair rising, *CMJ* counter movement jump, *SPPB* Short Physical Performance Battery Test, *cm* centimetre, *mm* millimetre, *N* newton; *N/s* Newton/seconds, *Nm* Newton-metre, *Nm/kg* Newton-meter/kilogram, *N/ms* Newton/milliseconds, *kg* kilogram, *J* Joule, *W* watt, *W/kg* watt/kilogram

Table 2 shows the training parameters. All authors prescribed two to three WBV sessions per week. Intervention duration of six trials were lasting more than 1 year [37, 38, 44, 46, 59, 67]. The duration of ten trials [35, 36, 42, 43, 50, 55–58, 53] was between 6 months and 1 year. The other trials lasted less than 6 months while one study examined strength effects immediately after a single WBV intervention [20]. The training parameters such as amplitude, frequency and sets of WBV varied across all studies investigating sinusoidal WBV. In contrast, studies with SR-WBV were more homogenous.

**Table 2:** Overview of trainings parameter within each study

Study	Duration/(Session/per week)	Amplitude	Frequency	Sets, Duration, Rest between set
Vertically sinusoidal vibration				
Amaral et al. [78]	12 weeks/(3/week)	2–4 mm	30–40Hz	3 sets × 30–45 s.
Álvarez- Barbosa et al. [62]	8 weeks/(3/week)	4 mm	F: 30–35Hz	6–12 sets, 12–17 min total time, 45 s rest between set
Bautmans et al. [34]	6 weeks/(3/week)	2–5 mm	30–40 Hz	4 sets × 30–60 s, 30–60 s rest between set
Beck et al. [35]	32 weeks/(2/week)	0.3 g	30 Hz	15 min (1 session), no rest
Bogaerts et al. [37]	47 weeks/(3/week)	2.5–5 mm	30–40 Hz	4 sets sets × 30 s – 15 × 30 s, 15–30 s rest between set
Bogaerts et al. [38]	48 weeks mo/(3/week)	NA	NA	NA
Boegarts [36]	24 weeks/(3/week)	1.6–2.2 g	30–40 Hz	3 sets × 15–60 s, 60–5 s rest between set
Corrie et al. [65]	12 weeks/(3/week)	1.3 mm	30 Hz	3 to 6 sets × 20 to 60 s, 60 s rest between set
Gomez-Cabello [42]	44 weeks mo/(3/week)	2 mm	40 Hz	10 sets × 45 s, 60 s rest between set
Kemmler et al. [44]	88 weeks/(2/week)	NA	25–35 Hz	NA
Kennis et al. [45]		2.5–5 mm	30–40 Hz	4 sets × 30 s till 15 sets × 30 s, 15–30 s rest between set
Klarner et al. [46]	48 weeks mo/(3/week)		35 Hz	7 sets × 90 s, 40 s rest between set
Lachance [47]	8 weeks/(2/week)	2 mm	35 Hz	NA
Leung et al. [66]	72 weeks mo/(5/week)	2 mm	35 Hz	20 min, rest (NA)
Machado et al. [48]	10 week/(3–5/week)	2–4 mm	20–40 Hz	3–8 sets × 30–60 s, rest (NA)
Mikhael et al. [49]	12 weeks/(3/week)	1 mm	12 Hz	10 sets × 60 s, 60 s rest between set
Roelants et al. [53]	24 weeks/(3/week)	2.5–5 mm	35–40 Hz	1–3 sets × 30–60 s of one exercise, 60 to 5 s rest between set
Sitjà-Rabert et al. [64]	6 weeks/(3/week)	2–4 mm	30–35 Hz	3 sets × 30–60 s of one exercise, 60 to 5 s rest between set
Verschueren et al. [58]	24 weeks/(3/week)	1.7–2.5 mm	35–40 Hz	NA
Verschueren et al. [57]	18 weeks/(3/week)	1.6–2.2 g	30–40 Hz	15–60 s × Pos. exercise, 60 s till 5 min rest between exercises
Sidealternating sinusoidal vibration				
Beck et al. [35]	32 weeks/(2/week)	2 mm	12.5 Hz	2 sets × 3 min, 60 s rest between set
Calder et al. [39]	6 weeks	2 mm	20 Hz	4 sets × 75 s, 90 s rest between set
Corrie et al. [65]	12 weeks/(3/week)	2.9 mm	30 Hz	3 to 6 sets × 20 to 60 s, 60 s rest between set
Furness and Maschette [40]	6 weeks	0.05 mm	15–25 Hz	5 sets × 60 s, 60 s rest between set
Furness et al. [41]	6 weeks/(3/week)	1 mm	15–25 Hz	5 sets × 60 s, 60 s rest between sets
Iwamoto et al. [43]	18 weeks/(2/week)	NA	20 Hz	4 min, NA rest
Klarner et al. [40]	48 weeks/(3/week)	3–7 mm	12,5 Hz	7 sets × 90 s, 40 s rest between set
Ochi et al. [67]	12 weeks/(3/week)	12 mm	2,5 Hz	180 s, no rest
Raimundo et al. [50]	32 weeks/(3/week)	6 mm	20–30 Hz	3 sets × 60 s, 60 s rest between set
Rees et al. [51]	8 weeks/(3/week)	5–8 mm	26 Hz	6 sets × 45 up to 60 s, 5 × 45 up to 80 s rest between set
Rees et al. [52]	8 weeks/(3/week)	5–8 mm	26 Hz	6 sets × 45–80 s, 45–80 s rest between set
Russo et al. [55]	24 weeks/(2/week)	NA	12–28 Hz	3 sets × 60–120 s, 60 s rest between set
Sievänen et al. [61]	10 week/(2/week)	2–8 mm	12 and 18 Hz	1–5 sets × 60–120 s, 60 s rest between set
Stolzenberg et al. [56]	36 weeks/(2/week)	NA	22–26 Hz	60–90 s, rest (NA)
von Stengel et al. [59]	74 weeks/(2weeks)	1.7–2 mm	25–35 Hz	6 sets × 60 s, 60 s rest between set
Zhang et al. [60]	8 weeks/(3–5/week)	1–3 mm	25–35 Hz	4–5 sets × 60 s, 60 s rest between set
Stochastic resonance vibration				
Kessler et al. [23]	4 weeks/(3/week)	–	3–6 Hz (Noise 4)	5 sets × 60 s, 60 s rest between set
Rogan et al. [54]	4 weeks/(3/week)	–	5 Hz (Noise 4)	5 sets × 60 s, 60 s rest between set
Rogan et al. [20]	Immediately (acute effects)		6 Hz (Noise 4)	5 sets × 60 s, 60 s rest between set

*Abbreviation*: *mo* month, *wk* week, *s* seconds, *Hz* hertz, *NA* not available

### Study methodological quality

**Fig. 2:**
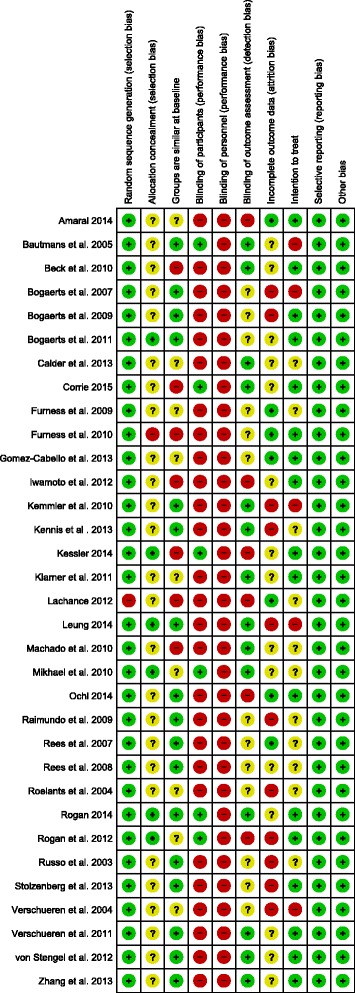
Risk of bias

All studies included in Fig. 2 were at risk of bias according to the “Cochrane Collaboration’s tool for assessing risk of bias”. Most studies lacked allocation concealment, blinding, and presented incomplete data.

### Meta-analysis

For the meta-analysis 37 studies were included and data were available for IMVC, DS, power, RFD or FS outcome measurements. The effect sizes for these outcomes are summarized in Figs. 3, 4, 5, 6, 7, 8, 9, 10, 11, 12 and 13.

**Fig. 3:**
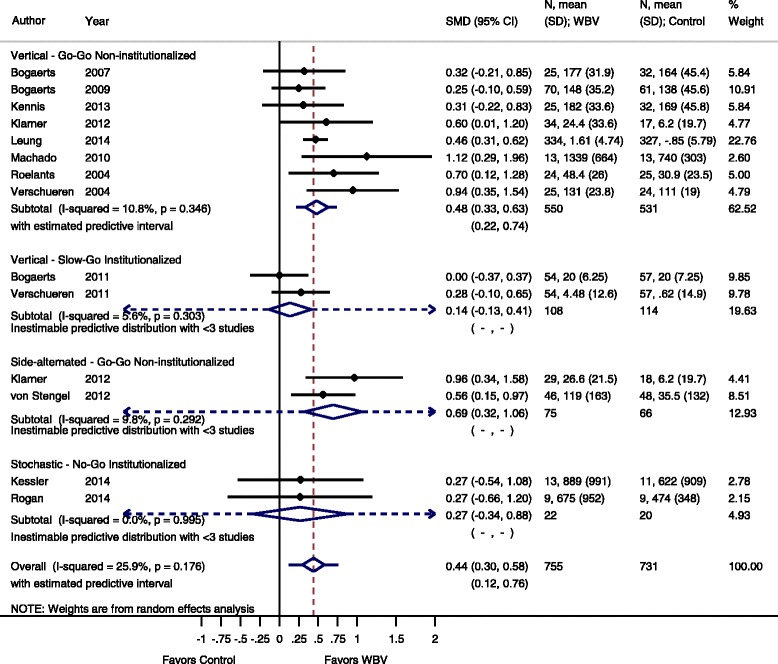
Comparison of WBV versus control group (i.e. no exercise), outcome: maximal voluntary isometric contraction. *SMD* standardized mean difference, *SD* standard deviation, *95 % CI* confidence interval, *I* ^*2*^ statistic for heterogeneity, *WBV* whole-body vibration

**Fig. 4:**
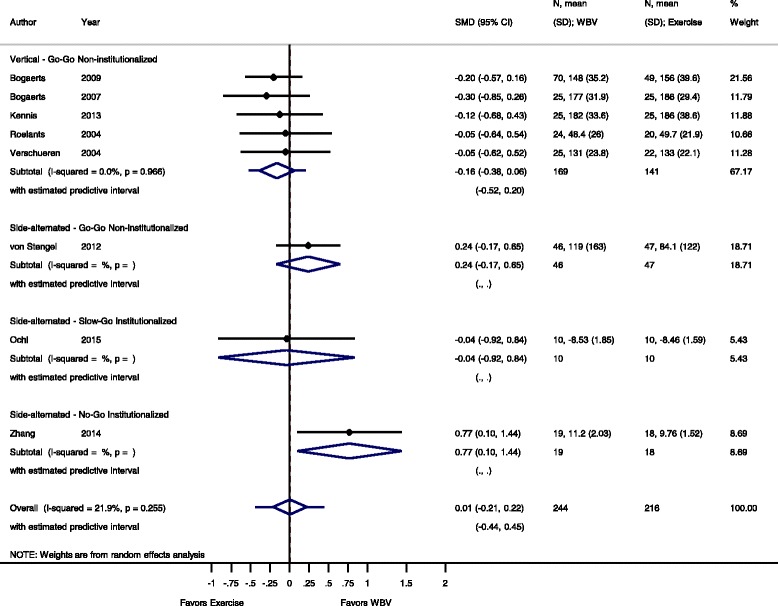
Comparison of WBV versus exercise group (i.e. no exercise), outcome: maximal voluntary isometric contraction. *SMD* standardized mean difference, *SD* standard deviation, *95 % CI* confidence interval, *I* ^*2*^ statistic for heterogeneity, *WBV* whole-body vibration

**Fig. 5:**
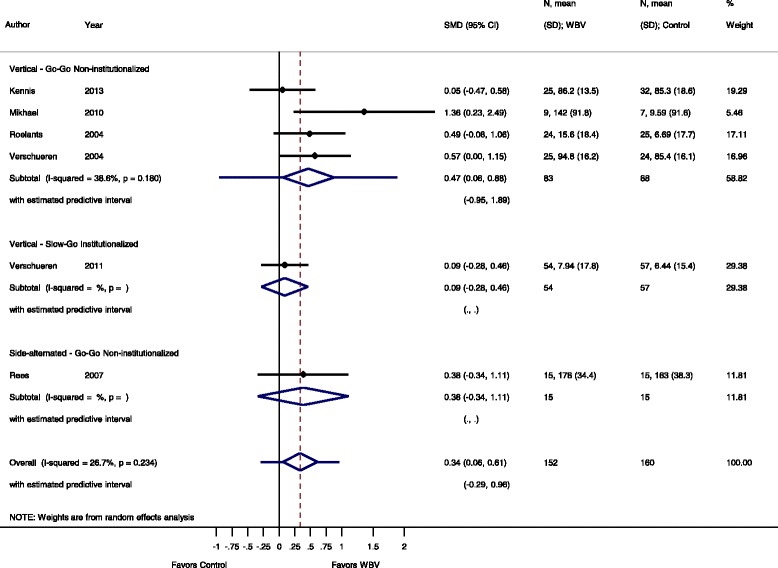
Comparison of WBV versus control group (i.e. no exercise) outcome: dynamic strength. *SMD* standardized mean difference, *SD* standard deviation, *95 % CI* confidence interval, *I* ^*2*^ statistic for heterogeneity, *WBV* whole-body vibration

**Fig. 6:**
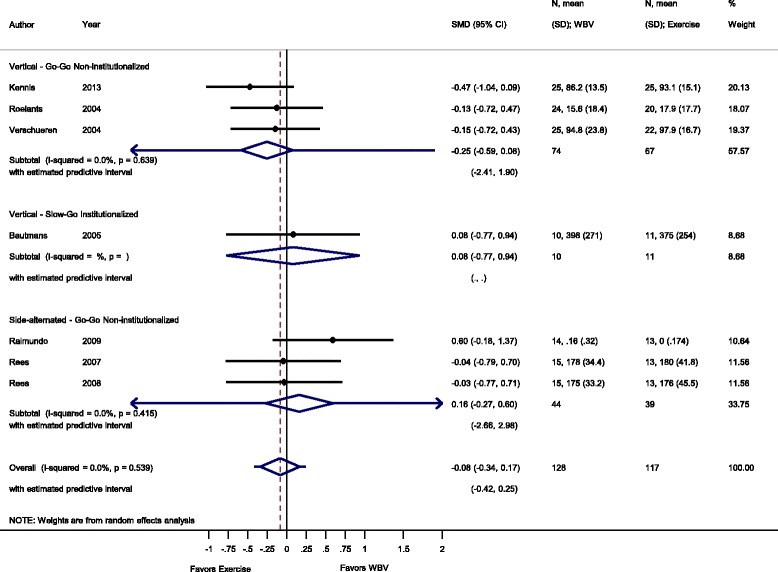
Comparison of WBV versus exercise group; outcome: dynamic strength. *SMD* standardized mean difference, *SD* standard deviation, *95 % CI* confidence interval, *I* ^*2*^ statistic for heterogeneity, *WBV* whole-body vibration

**Fig. 7:**
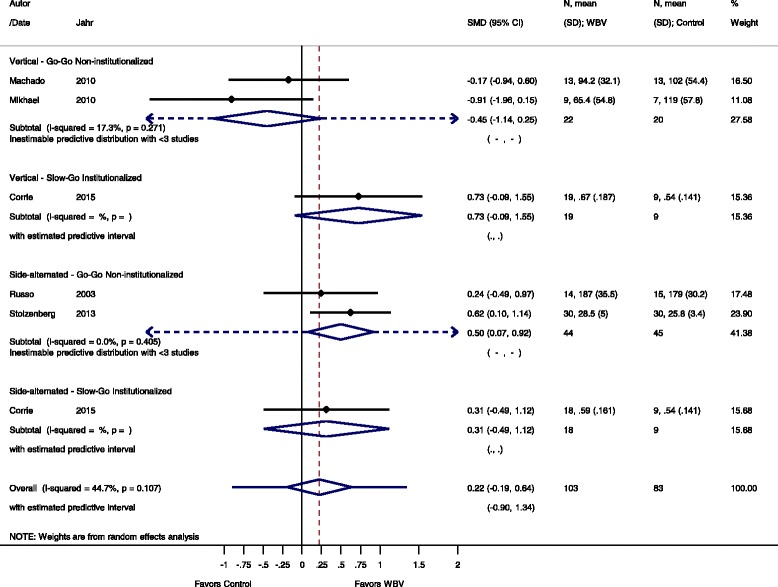
Comparison of WBV versus control group (i.e. no exercise) outcome: power. *SMD* standardized mean difference, *SD* standard deviation, *95 % CI* confidence interval, *I* ^*2*^: statistic for heterogeneity, *WBV* whole-body vibration

**Fig. 8:**
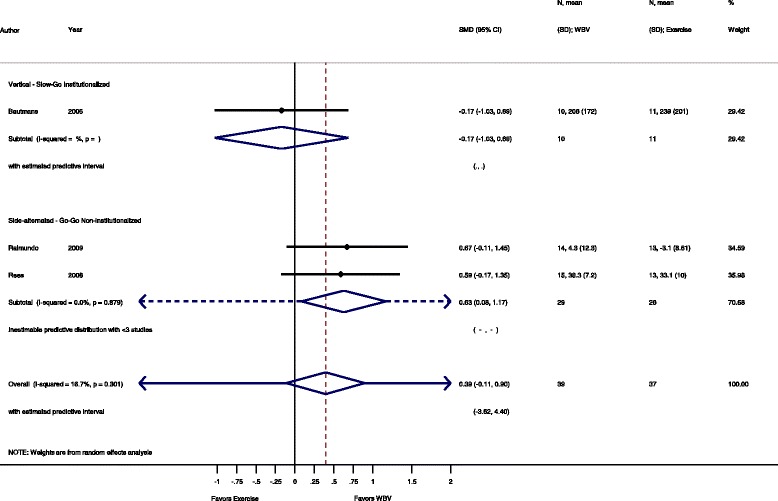
Comparison of WBV versus exercise group; outcome: power. *SMD* standardized mean difference, *SD* standard deviation, *95 % CI* confidence interval, *I* ^*2*^ statistic for heterogeneity, *WBV* whole-body vibration

**Fig. 9:**
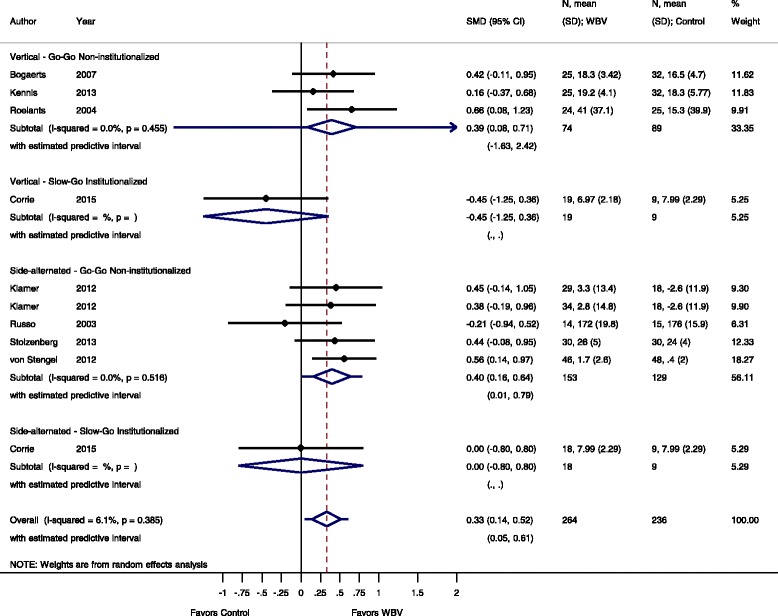
Comparison of WBV versus control group (i.e. no exercise), outcome: rate of force development. *SMD* standardized mean difference, *SD* standard deviation, *95 % CI* confidence interval, *I* ^*2*^ statistic for heterogeneity, *WBV* whole-body vibration

**Fig. 10:**
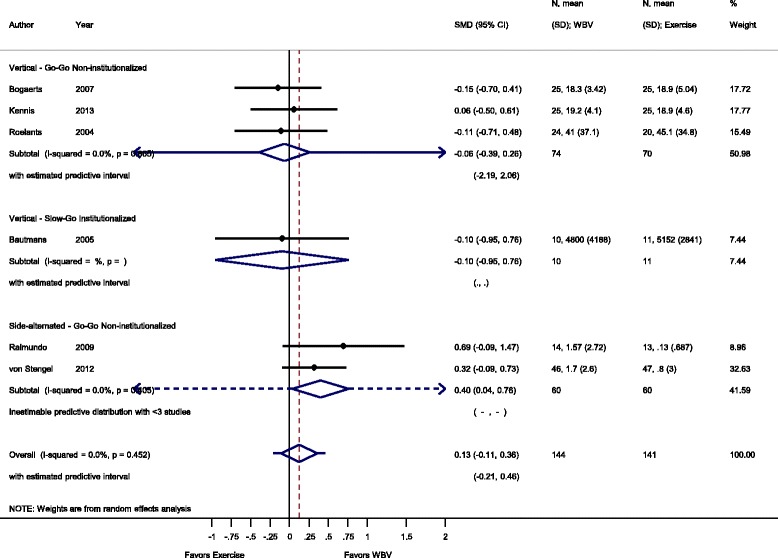
Comparison of WBV versus exercise group, outcome: rate of force development. *SMD* standardized mean difference, *SD* standard deviation, *95 % CI* confidence interval, *I* ^*2*^ statistic for heterogeneity, *WBV* whole-body vibration

**Fig. 11:**
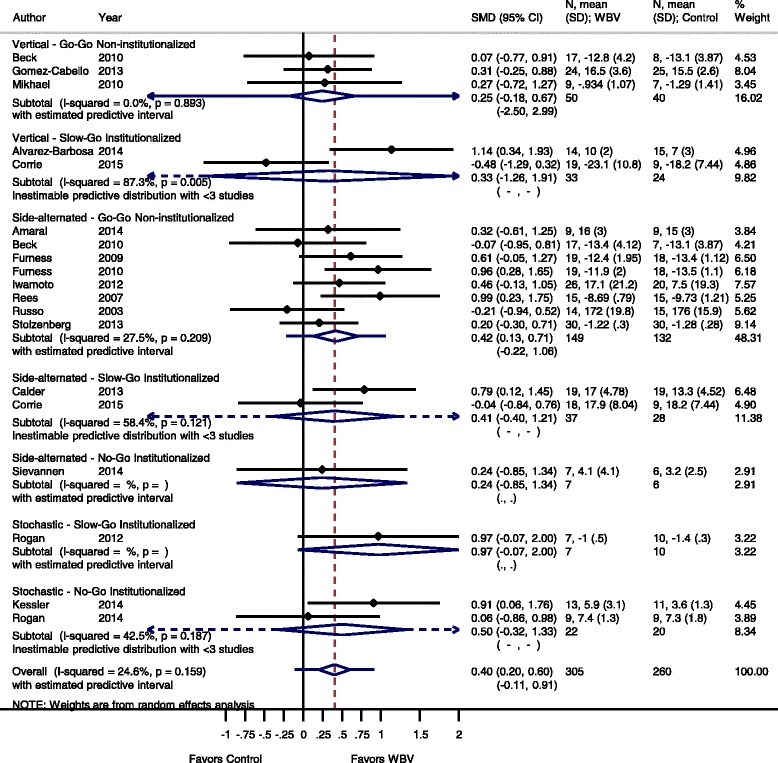
Comparison of WBV versus control group (i.e. no exercise), outcome: functional strength. *SMD* standardized mean difference, *SD* standard deviation, *95 % CI* confidence interval, *I* ^*2*^ statistic for heterogeneity, *WBV* whole-body vibration

**Fig. 12:**
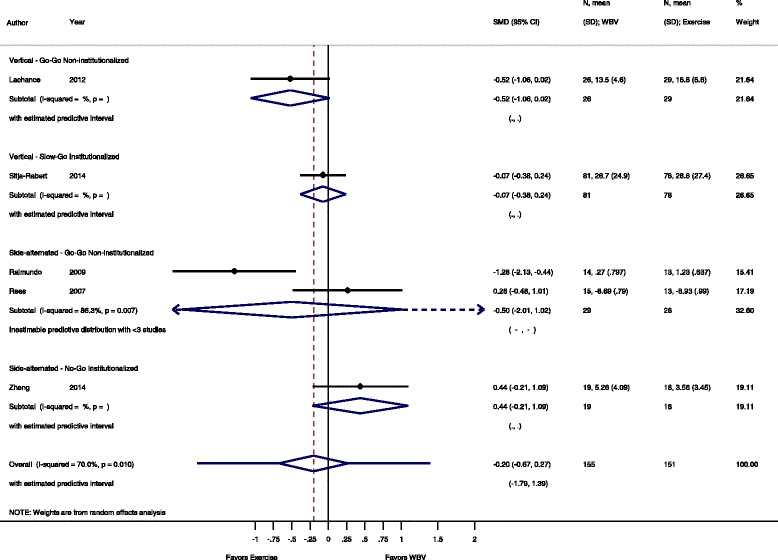
Comparison of WBV versus exercise group, outcome: functional strength. *SMD* standardized mean difference, *SD* standard deviation, *95 % CI* confidence interval, *I* ^*2*^ statistic for heterogeneity, *WBV* whole-body vibration

**Fig. 13:**
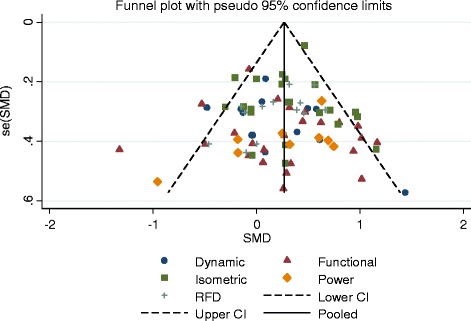
Funnel plot over all analysis

#### Isometric maximal voluntary contraction: WBV vs. non-exercise control group

Thirteen studies [20, 23, 36–38, 45, 46, 48, 53, 57–59, 67], including 1468 participants, reported data contributing to the comparison WBV vs. non-exercise control group, one study had two WBV-arms (vertical and side-alternating) and one control arm [46] (the number of participants in the control groups was cut in half to obtain correct numbers for the pooled analysis). The pooled overall SMD was 0.44 (95 % CI 0.30 to 0.58) in favor of WBV with low heterogeneity, I^2^ 25.9 % (*p* = 0.176).

#### Go-Go

The subgroup analysis for SV-WBV-Go-Go showed a SMD of 0.48 (95 % CI 0.33 to 0.63) with a low heterogeneity (I^2^ 10.8 % (*p* = 0.346)); and for SS-WBV-Go-Go a SMD of 0.69 (95 % CI 0.32 to 1.06) with a low heterogeneity, I^2^ 9.8 % (*p* = 0.292).

#### Slow-Go

The subgroup analysis for SV-WBV-Slow-Go revealed a SMD of 0.14 (95 % CI −0.13 to 0.41) with a low heterogeneity, I^2^ 5.6 % (*p* = 0.303).

#### No-Go

The subgroup analysis for SR-WBV-No-Go showed a SMD of 0.27 (95 % CI −0.34 to 0.88) in favour of SR-WBV with no heterogeneity, I^2^ 0.0 % (*p* = 0.995) (Fig. 3).

#### Isometric maximal voluntary contraction: WBV vs. exercise group

For the comparison WBV versus conventional exercise eight studies [37, 38, 45, 53, 58–60, 68] contributed 460 participants. The pooled overall SMD was 0.01 (95 % CI −0.21 to 0.22) with a low heterogeneity, I^2^ = 21.9 % (*p* = 0.255).

#### Go-Go

The subgroup analysis for SV-WBV-Go-Go showed a SMD of −0.16 (95 % CI −0.38 to 0.06) with a low heterogeneity, I^2^ 0.0 % (*p* = 0.966); SS-WBV-Go-Go showed an SMD of 0.24 (95 % CI −0.17 to 0.65) in favour of SS-WBV.

#### Slow-Go

The subgroup analysis for SS-WBV-Slow-Go showed a SMD of −0.04 (95 % CI −0.92 to 0.84) in favour of conventional exercise.

#### No-Go

The subgroup analysis for SS-WBV-No-Go showed a SMD of 0.77 (95 % CI 0.10 to 1.44) in favour of SS-WBV.

#### Dynamic strength: WBV vs. non-exercise control

Comparing dynamic strength in WBV versus non-exercise controls, six studies [45, 49, 51, 53, 57, 58] contributed with a total of 312 participants. The pooled overall SMD was 0.34 (95 % CI 0.06 to 0.61), which was statistically significant in favour of the WBV group, with low heterogeneity (I^2^ 26.7 %, *p* = 0.234).

#### Go-Go

The subgroup analysis for SV-WBV-Go-Go showed a SMD of 0.47 (95 % CI 0.06 to 0.88) in favour of SV-WBV with a low heterogeneity, I^2^ 38.6 %, *p* = 0.180. The subgroup analysis for SS-WBV-Go-Go showed a SMD of 0.38 (95 % CI −0.34 to 1.11) in favour of SS-WBV.

#### Slow-Go

The subgroup analysis for SV-WBV-Slow-Go showed a SMD of 0.09 (95 % CI −0.28 to 0.46).

#### Dynamic strength: WBV vs. conventional exercise

For the comparison WBV versus conventional exercise seven studies [45, 50–52, 58, 59, 53] contributed with a total of 245 participants. The pooled overall SMD was 0.08 (95 % CI −0.34 to 0.17), statistically non-significant, thus, not in favour of a particular group, with no heterogeneity (I^2^ 0.0 %, *p* = 0.539).

#### Go-Go

The subgroup analysis for SV-WBV-Go-Go showed a SMD of −0.25 (95 % CI −0.59 to 0.08) in favour of exercise, with no heterogeneity, I^2^ 0.0 % (*p* = 0.639). The subgroup analysis for SS-WBV-Go-Go showed a SMD of 0.16 (95 % CI −0.27 to 0.60) statistically not significant in favour of SS-WBV with no heterogeneity, I^2^ 0.0 % (*p* = 0.415).

#### Slow-Go

The subgroup analysis for SV-WBV-Slow-Go showed a SMD of 0.08 (95 % CI −0.77 to 0.94).

#### Power: WBV vs. non-exercise control

For the outcome power and the comparison WBV versus non-exercise control five studies [48, 49, 55, 56, 66] contributed with a total of 186 participants. The control group of the Corrie et al. [65] study contributed to two subgroup analyses, therefore, we cut the number of participants in the control group in half to avoid incorrect standard errors.

#### Go-Go

The pooled overall SMD was 0.22 (95 % CI −0.19 to 0.64), statistically non-significant in favour of the WBV group, with a moderate heterogeneity (I^2^ 44.7 %, *p* = 0.107).

The subgroup analysis for SV-WBV-Go-Go showed a SMD of −0.45 (95 % CI −1.14 to 0.25), with low heterogeneity, I^2^ 17.3 % (*p* = 0.271). SS-WBV-Go-Go showed a SMD of 0.50 (95 % CI 0.07 to 0.92) statistically significant in favour of SS-WBV with no heterogeneity, I^2^ 0.0 % (*p* = 0.405).

#### Slow-Go

The subgroup analysis for SV-WBV-Slow-Go showed a SMD of 0.73 (95 % CI −0.09 to 1.55). SS-WBV-Slow-Go showed a SMD of 0.31 (95 % CI −0.49 to 1.12) statistically not significant in favour of SS-WBV.

#### Power: WBV vs. conventional exercise

Three studies [34, 50, 52] with 76 participants contributed to the comparison SS-WBV versus conventional exercise. The SMD was 0.39 (95 % CI −0.11 to 0.90), statistically not significant in favour of the WBV group.

#### Go-Go

The subgroup analysis for SS-WBV-Go-Go showed a SMD of 0.63 (95 % CI 0.08 to 1.17), statistically significant in favour of SS-WBV with no heterogeneity, I^2^ 0.0 % (*p* = 0.879).

#### Slow-Go

The subgroup analysis for SV-WBV-Slow-Go showed a SMD of −0.17 (95 % CI −1.03 to 0.69), statistically not significant in favour of conventional exercise.

#### Rate of force development: WBV vs. non-exercise control

For the outcome rate of force development and the comparison WBV versus non-exercise control eight studies [34, 37, 45, 46, 55, 56, 66, 53] contributed with a total of 500 participants. The control groups of two studies [46, 66] contributed to two subgroup analyses, therefore, we divided the number of participants in the control groups in half. The pooled overall SMD was 0.33 (95 % CI 0.14 to 0.52), statistically significant in favour of the WBV group with low heterogeneity (I^2^ 6.1 %, *p* = 0.385).

#### Go-Go

The subgroup analysis for SV-WBV-Go-Go showed a SMD of 0.39 (95 % CI 0.08 to 0.71) in favour of conventional exercise with no heterogeneity, I^2^ 0.0 % (*p* = 0.455). SS-WBV-Go-Go showed a SMD of 0.40 (95 % CI 0.16 to 0.64) and statistically significant in favour of SS-WBV with no heterogeneity, I^2^ 0.0 % (*p* = 0.516).

#### Slow-Go

The subgroup analysis for SV-WBV-Slow-Go showed a SMD of −0.45 (95 % CI −1.25 to 0.36). The subgroup analysis for SS-WBV-Slow-Go showed a SMD of 0.00 (95 % CI −0.80 to 0.80).

#### Rate of force development: WBV vs. conventional exercise

For the comparison WBV versus conventional exercise six studies [34, 37, 45, 50, 59, 53] contributed with a total of 285 participants. The pooled overall SMD was 0.13 (95 % CI −0.11 to 0.36), statistically non-significant in favour of the WBV group, with no heterogeneity (I^2^ 0.0 %, *p* = 0.452).

#### Go-Go

The subgroup analysis for SV-WBV-Go-Go showed a SMD of −0.06 (95 % CI −0.39 to 0.26) in favour of exercise, with no heterogeneity, I^2^ 0.0 % (*p* = 0.805). SS-WBV-Go-Go showed a SMD of 0.40 (95 % CI 0.04 to 0.76), statistically significant in favour of SS-WBV with no heterogeneity, I^2^ 0.0 % (*p* = 0.405).

#### Slow-Go

The subgroup analysis for SV-WBV-Slow-Go showed a SMD of −0.10 (95 % CI −0.95 to 0.76).

#### Functional strength: WBV vs. non-exercise control

For the outcome functional strength and the comparison WBV versus non-exercise control 17 studies [20, 23, 35, 37, 39–43, 45, 49, 51, 54–56, 59, 64, 53] contributed with a total of 565 participants (one study has SS-WBV and SV-WBV versus control, therefore we cut the number of participants of the control group in half) [35]. The pooled overall SMD was 0.40 (95 % CI 0.20 to 0.60), statistically non-significant in favour of the WBV group with low heterogeneity (I^2^ 24.6 %, *p* = 0.159).

#### Go-Go

The subgroup analysis for SV-WBV-Go-Go showed an SMD of 0.25 (95 % CI −0.189 to 0.67), statistically non-significant in favour of exercise with no heterogeneity, I^2^ 0.0 % (*p* = 0.893).SS-WBV-Go-Go showed a SMD of 0.42 (95 % CI 0.13 to 0.71) statistically significant in favour of SS-WBV with low heterogeneity, I^2^ 27.5 % (*p* = 0.209).

#### Slow-Go

The subgroup analysis for SV-WBV-Slow-Go showed an SMD of −0.33 (95 % CI −1.26 to 1.91) with high heterogeneity, I^2^ 87.3 % (*p* = 0.005). The subgroup analysis for SS-WBV-Slow-Go showed a SMD of 0.41 (95 % CI −0.40 to 1.21), statistically non-significant in favour of SS-WBV with moderate heterogeneity, I^2^ 58.4 % (*p* = 0.121).

#### No-Go

The subgroup analysis for SS-WBV-No-Go showed a SMD of 0.24 (95 % CI −0.85 to 1.34). The subgroup analysis for SR-WBV-Slow-Go showed a SMD of 0.97 (95 % CI −0.07 to 2.00). The subgroup analysis for SR-WBV-No-Go showed a SMD of 0.50 (95 % CI −0.32 to 1.33), statistically non-significant in favour of SR-WBV, with a moderate heterogeneity, I^2^ 42.5 % (*p* = 0.187).

#### Functional strength: WBV vs. conventional exercise

For the comparison WBV versus conventional exercise five studies [47, 50, 51, 60, 64] contributed with a total of 306 participants. The pooled overall SMD was −0.20 (95 % CI −0.67 to 0.27), statistically non-significant in favour of the conventional exercise group with substantial heterogeneity (I^2^ 70.0 %, *p* = 0.010).

#### Go-Go

The subgroup analysis for SV-WBV-Go-Go showed a SMD of −0.52 (95 % CI −1.06 to 0.02), statistically non-significant in favour of conventional exercise. The subgroup analysis for SS-WBV-Go-Go showed a SMD of −0.50 (95 % CI −2.01 to 1.02) with high heterogeneity, I^2^ 86.3 % (*p* = 0.007).

#### Slow-Go

The subgroup analysis for SV-WBV-Slow-Go showed a SMD of −0.07 (95 % CI −0.38 to 0.24).

#### No-Go

The subgroup analysis for SS-WBV-No-Go showed a SMD of 0.44 (95 % CI −0.21 to 1.09), statistically non-significant in favour of SS-WBV.

Over all comparisons, only the comparisons of WBV versus non-exercise control for the outcomes isometric strength had a significant Egger’s test (*p* = 0.002) for small study bias. Funnel plots for individual comparison are not shown. Figure 13 shows a panel of the funnel plots for all comparisons.

The effect sizes in the studies with participants in the No-Go group (SMD of 0.47, 95 % CI 0.16 to 0.78, I^2^ 0.00 %, *p* <0.792) were higher compared to the Go-Go (SMD 0.26, 95 % CI 0.15 to 0.63, I^2^ 48.9 %, *p* <0.001) and the Slow-Go groups (SMD of 0.14, 95 % CI −0.04 to 0.33, I^2^ 26.6 %, *p* = 0.121) (see Table 3 and Additional files 2, 3 and 4).

**Table 3:** Overview of WBV utilization on physical performance status

	Go-Go		Slow-Go		No-Go
	VS-WBV		VS-WBV		
1	Amaral et al. [78] (SA) FS	1	Alvarez et al. [62] FS		
2	Beck et al. [35] FS	2	Bautmans [34] DS, Power, RFD		
3	Boegarts et al. [37] IMVC, RFD	3	Boegarts et al. [36] IMVC		
4	Boegarts et al. [38] IMVC	4	Corrie et al. [6] Power, FS, RFD		
5	Gomez-Cabello et al. [42] FS	5	Sitja-Rabert et al. [68] FS		
6	Kemmler et al. [44] IMVC, Power,	6	Verscheuern et al. [57] IMVC, DS		
7	RFD				
8	Kennis et al. [45] IMVC, DS, RFD				
9	Klarner et al. [46] IMVC, RFD				
10	Lachane [47] FS				
11	Leung et al. [66] IMVC				
12	Machado et al. [48] MVC, Power				
13	Mikhael et al. [49] DS, Power, FS				
14	Roelants et al. [53] IMVC, DS, RFD				
15	Verscheuren et al. [58] IMVC, DS				
	SS-WBV		SS-WBV		SS-WBV
1	Beck et al. [35] FS	1	Calder et al. [39] FS	1	Sievänen et al. [61] FS
2	Furness and Maschette [40] FS	2	Corrie et al. [65] Power, RFD, FS	2	Zhang et al. [60] IMVC, FS
3	Furness et al. [41] FS	3	Ochi et al. [67] IMVC		
4	Iwamoto et al. [43] FS				
5	Klarner et al. [46] IMVC, RFD				
6	Raimundo et al. [50] DS, Power, RFD; FS				
7	Rees et al. [51] DS, FS				
8	Rees et al. [52] DS, Power				
9	Russo et al. [55] DS, RFD				
10	Stolzenberg et al. [56] Power, RFD, FS				
11	von Stengel et al. [59] IMVC, RFD				
			SR-WBV		SR-WBV
1		1	Rogan et al. [54] FS	1	Kessler et al. [23] IMVC, RFD, FS
2				2	Rogan et al. [20] IMVC, IRFD, FS
	SMD 0.26 (95 % CI 0.15 to 0.63)		SMD of 0.14 (95 % CI −0.04 to 0.33)		SMD of 0.47 (95 % CI 0.16 to 0.78)
	I^2^ 48.9 %, *p* <0.001		I^2^ 26.6 %, *p* = 0.121		I^2^ 0.00 %, *p* <0.792

*Abbreviation: VS-WBV* vertical sinusoidal whole-body vibration, *SS-WBV* side-alternating whole-body vibration; *SR-WBV* stochastic resonance whole-body vibration, *IMVC* isometric maximal voluntary contraction, *DS* dynamic maximal strength, *RFD* rate of force development, *IRFD* isometric rate of force development, *FS* functional strength, SMD, *I* ^*2*^ I^2^ –statistic for heterogeneity

## Discussion

We hypothesized that WBV differently effects on measures of strength and power in Go-Go, Slow-Go and No-Go. This systematic review on muscle-strength-related outcomes of WBV in healthy elderly participants included 37 studies in a final analysis, most of which were studies with small sample sizes. The main findings were that WBV showed low to moderate effects in Go-Go, Slow-Go and No-Go when compared to non-exercising control groups on proxies of muscle strength in older adults. Furthermore, compared to groups performing more conventional types of exercise, WBV had only small and mostly non-significant advantages. Although only a few studies evaluated the effects of WBV in samples of elderly participants in need of care (classified as No-Go [10] in this review), the highest effect sizes favouring WBV were found in these studies. The few studies that evaluated SR-WBV also resulted in high effect sizes in favour of this type of WBV in No-Go. These findings seem to confirm our hypothesis. However, when we summarized the effect sizes of the meta-analysis and intended to perform statistical analysis on these data that would either refute or confirm our hypothesis, the amount of data was not big enough and the groups too unbalanced in size to allow a credible analysis to be performed. Further studies in No-Go are, therefore, warranted and needed with the various types of WBV. This means regarding our aim to give recommendations on available evidence for practical use the inference must be that at present no recommendations can be given for the most effective vibration mode in elderly persons.

Our review classified the physical capacities of the included participants (i.e. in “Go-Go”, “Slow-Go”, and “No-Go” [10]) and analysed studies with WBV versus non-exercising control separately from studies comparing WBV versus other types of conventional strength training exercise, as recommended by Orr [68]. Furthermore, we separately analyzed studies using vertical, side-alternating and stochastic resonance WBV. The reason for these distinctions relate to the assumption that initial fitness when entering a training program together with the training content may differently effect on training outcomes. Physical fitness includes health-related (cardiorespiratory endurance, muscular endurance, muscular strength, flexibility and body composition) and skill-related components (agility, coordination, balance, speed, reaction time and power) [69]. Although exercise recommendations have been published for older adults; e.g. the American College of Sports Medicine (ACSM) [70] guidelines recommend that older adults should undertake 30 min of moderate intensity, aerobic exercise or activity, five times per week to incur any health benefits, the complex interactions present in various sub-populations of older adults preclude the definition of specific, detailed exercise prescriptions. Furthermore, the number of older people fulfilling the ACSM requirement is rather small and most likely even lower amongst those with low levels of functioning [71]. It seems fair to assume that when principles of exercise training are applied to the development of exercise protocols, clinicians in practical settings can have greater confidence that non-significant research findings reflect deficiencies in exercise efficacy rather than deficiencies in exercise prescription [72]. It is thereby important, however, to consider low baseline fitness and mobility levels in pre-frail or frail or rather untrained elderly when starting an exercise program. Based on the findings of this systematic review it seems that the use of (SR)-WBV is valuable for untrained or frail elderly where the neuromuscular systems might not be able withstanding higher loading and long training sessions, however, with increasing levels of functioning there is a diminishing effect of the WBV interventions. Considering this it becomes clear that this systematic review only reveals first estimates for the possible effect of WBV in (pre-)frail elderly. An important next step would be the design and implementation of a sufficiently powered WBV exercise study specifically targeting (pre-)frail institutionalised elderly with a training duration of at least 2 months since this is the duration where effects of WBV training in the elderly may be expected [73].

### Role of physical capacity level

Altough we did not find a systematic review on WBV that used a classification of participants in different subgroups of functioning, our conclusion is in line with similar previously expressed conclusions. Lau et al. [24] mainly focussed on bone mineral density but also included strength measures. They concluded, that WBV is beneficial in elderly persons to increase muscle strength. However, because they did not use a separate analysis for different groups with different levels of physical capacities it is difficult to determine whether the effects observed are dependent on the baseline fitness of the study participants. Sitja-Rabert et al. [25] concluded that WBV was beneficial in elderly participants to improve strength. Osawa et al. [74] included both young and elderly participants and concluded that WBV in addition to exercises or to a normal lifestyle improved knee extensor strength and countermovement jump performance when compared to identical training conditions without WBV.

### WBV as skilling-up exercise

Our findings seem to justify the assumption, that WBV might be applicable as a “skilling-up” exercise for elderly with low physical capacity (i.e. the No-Go group), who are considering to begin with an exercise program, but who are not yet able to perform traditional strength exercises. A short bout of vibration would produce sufficient stimuli to effect on muscle strength, power and functional tasks. There are some theoretical explanations that WBV might improve the neuromuscular drive, which improves muscular function [18, 75]. In contrary to the No-Go group, elderly persons in the Go-Go and the Slow-Go group can perform standard exercises and WBV might be an additional option among all exercise modalities. The measured effects sizes for the Go-Go and Slow-Go groups were not very high and not all outcomes showed significant differences in the included studies. For this reason, WBV should be rather used for “skilling-up” in pre-frail or frail elderly individuals.

### Limitations of this study

There are some limitations of this review. The included studies presented moderate to high risk of bias, allocation concealment was not described in most studies, groups were often not similar at baseline (which is often the case in small studies), participants were blinded in only seven studies and outcome assessors were blinded in only 11 studies. With the exception of Leung et al. [66], Boegarts et al. [38], Kemmler et al. [44], von Stengel [59] (*n* = 151) and Sitjà-Rabert et al. [64], the remaining studies were small. This further increases the risk of bias. Therefore, the quality of the included studies overall was rather low.

The ability to replicate or reproduce experimental results, or reproducibility, is one of the major tenets of the scientific method. SR-WBV results considered in this review come from one research group only. It is, therefore, necessary that the published findings of this group are validated through replication by others. Until replication is done the results for SR-WBV should be interpreted with caution and, hence, this is a limitation of this review. Another limitation of our approach is the possible language bias, we only included studies in English or German and it cannot be excluded that relevant research in different languages exists.

This review included mainly studies with “Go-Go” and “Slow-Go” participants and only few studies with participants from the “No-Go” group. As the effect might be largest in this No-Go group with SMD of 0.47 (95 % CI 0.16 to 0.78), further studies should evaluate WBV in this group of elderly. Only a few studies have evaluated SR-WBV and no study has compared SR-WBV to other vibration modalities in elderly participants.

## Conclusions

WBV shows beneficial effects on proxies of muscle strength in older adults, mainly in elderly with lower initial levels of functioning, suggesting that WBV can be used as a skilling-up exercise in participants. However, the review suggests that WBV has no overall treatment effect on muscle strength properties in older women and men across the whole spectrum of physical functioning. Only few studies evaluated WBV in (pre-)frail elderly. No randomized trial has examined the effects of WBV on muscle in older (pre-)frail elderly. Based on this review no recommendations can be given for the most effective vibration mode. Further studies with the various types of WBV in various sub-populations of elderly persons are warranted and needed to determine the most effective vibration modes.

## Additional files

Additional file 1:
**Search terms in PubMed.** (DOCX 62 kb) Additional file 2:
**Forest plot overview of Classification Go-Go, outcome: all strength outcomes (IMVC, DS, Power, RFD, FS).** (PPTX 165 kb) Additional file 3:
**Forest plot overview of Classification Slow-Go, outcome: all strength outcomes (IMVC, DS, Power, RFD, FS).** (PPTX 105 kb) Additional file 4:
**Forest plot overview of Classification No-Go, outcome: all strength outcomes (IMVC, DS, Power, RFD, FS).** (PPTX 75 kb)

## Abbreviations

CENTRAL: Cochrane Central Register of Controlled Trials
DS: dynamic strength
FS: functional strength
Go-Go: independent person
IMVC: isometric maximum voluntary contraction
No-Go: person depending permanently on assistance with severe functional limitation
PEDro: Physiotherapy Evidence Database
RFD: rate of force development
Slow-Go: depending on support in everyday activities such as dressing, body care, eating, using the toilet, mobility, and planning the day
SMD: standardised mean differences
SR-WBV: stochastic resonance whole-body vibration
SS-WBV: side-alternating sinusoidal whole-body vibration
SV-WBV: sinusoidal vertical whole-body vibration
WBV: whole-body vibration
WHO: World Health Organization
