# Supplementary material for: Effects of whole-body vibration on proxies of muscle strength in old adults: a systematic review and meta-analysis on the role of physical capacity level
Source: Eur Rev Aging Phys Act. 2015 Dec 8;12:12. doi: 10.1186/s11556-015-0158-3 (PMC4748331; doi:10.1186/s11556-015-0158-3)
Supplement: Additional file 1: — Search terms in PubMed. (DOCX 62 kb) [file 11556_2015_158_MOESM1_ESM.docx]

Additional file 1

Serach terms in PubMed:

Search terms: ((((elderly) OR age) OR frail)) AND ((((((((((((((((((((((strength)) OR ((power))) OR ((rate of force development))) OR ((force))) OR ((functional strenght))) OR ((fall))) OR ((falls))) OR ((faller))) OR ((fallers))) OR ((sway))) OR ((postural reaction))) OR ((postural stability))) OR ((postural balance))) OR ((gait stability))) OR ((dynamic stability)))) OR ((propriocept*))) OR ((sensori-motor))) OR ((sensorimotor)))) AND ((("whole body vibration") OR ("whole body vibrations") OR (Vibration Training[tiab]) OR (stochastic stimulation) OR (stochastic training) OR (stochastic vibration) OR (Whole-Body Vibration) OR (Whole-Body-Vibration) OR (Whole-Body Vibrations) OR (Whole-Body-Vibrations) OR (WBV) OR (sinusoidal vibration) OR (noise vibration AND (therapy OR treatment OR training OR exercise)) OR (stochastic resonance therapy) OR (stochastic resonance treatment))))
