# Supplementary material for: Effects of whole-body vibration on proxies of muscle strength in old adults: a systematic review and meta-analysis on the role of physical capacity level
Source: Eur Rev Aging Phys Act. 2015 Dec 8;12:12. doi: 10.1186/s11556-015-0158-3 (PMC4748331; doi:10.1186/s11556-015-0158-3)
Supplement: Additional file 2: — Forest plot overview of Classification Go-Go, outcome: all strength outcomes (IMVC, DS, Power, RFD, FS). (PPTX 165 kb) [file 11556_2015_158_MOESM2_ESM.pptx]

## Slide 1
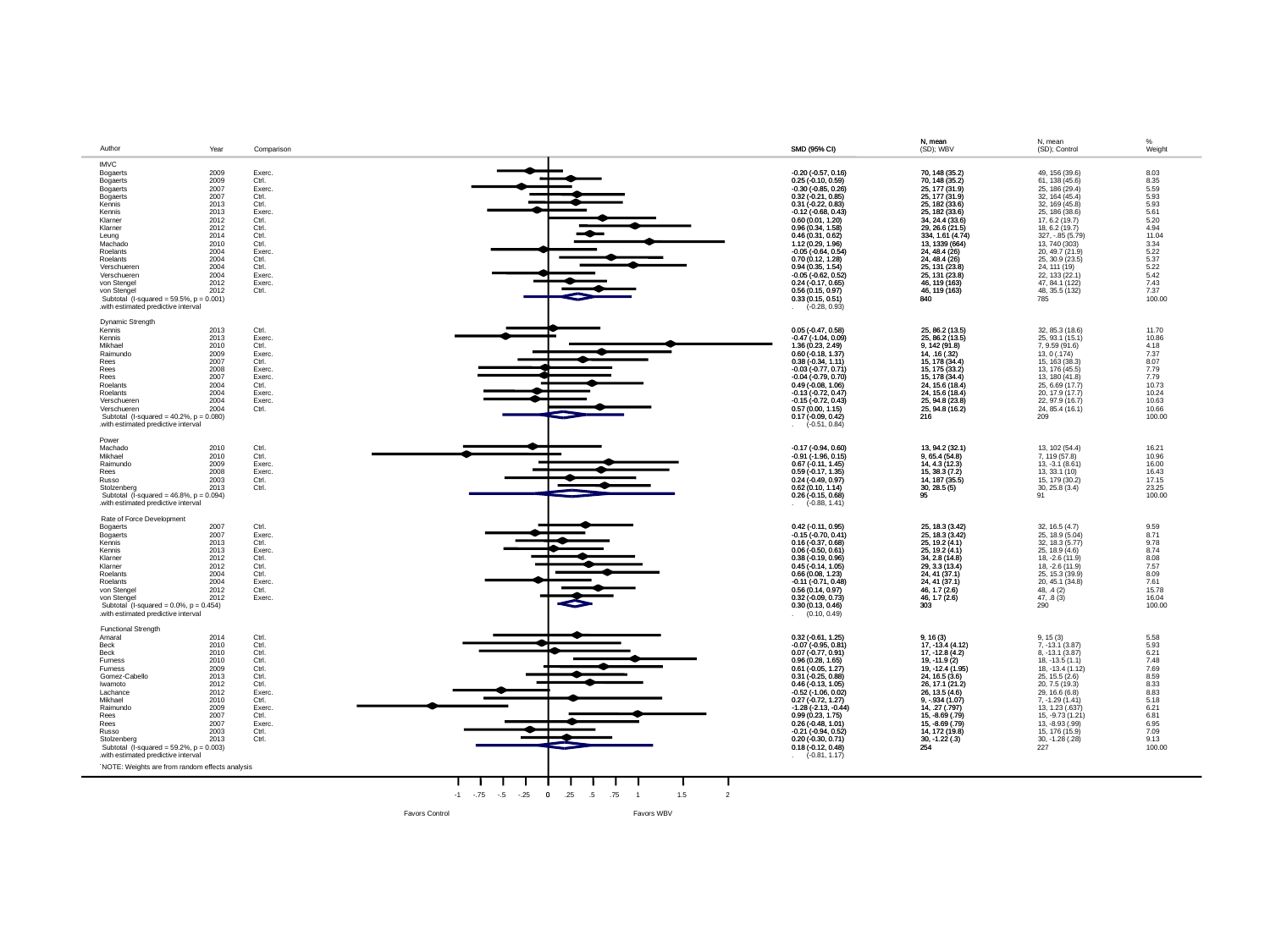

N, mean
N, mean
N, mean
%
Author
Year
Comparison
SMD (95% CI)
SMD (95% CI)
(SD); WBV
(SD); Control
Weight
IMVC
Bogaerts
2009
Exerc.
-0.20 (-0.57, 0.16)
-0.20 (-0.57, 0.16)
70, 148 (35.2)
70, 148 (35.2)
49, 156 (39.6)
8.03
Bogaerts
2009
Ctrl.
0.25 (-0.10, 0.59)
0.25 (-0.10, 0.59)
70, 148 (35.2)
70, 148 (35.2)
61, 138 (45.6)
8.35
Bogaerts
2007
Exerc.
-0.30 (-0.85, 0.26)
-0.30 (-0.85, 0.26)
25, 177 (31.9)
25, 177 (31.9)
25, 186 (29.4)
5.59
Bogaerts
2007
Ctrl.
0.32 (-0.21, 0.85)
0.32 (-0.21, 0.85)
25, 177 (31.9)
25, 177 (31.9)
32, 164 (45.4)
5.93
Kennis
2013
Ctrl.
0.31 (-0.22, 0.83)
0.31 (-0.22, 0.83)
25, 182 (33.6)
25, 182 (33.6)
32, 169 (45.8)
5.93
Kennis
2013
Exerc.
-0.12 (-0.68, 0.43)
-0.12 (-0.68, 0.43)
25, 182 (33.6)
25, 182 (33.6)
25, 186 (38.6)
5.61
Klarner
2012
Ctrl.
0.60 (0.01, 1.20)
0.60 (0.01, 1.20)
34, 24.4 (33.6)
34, 24.4 (33.6)
17, 6.2 (19.7)
5.20
Klarner
2012
Ctrl.
0.96 (0.34, 1.58)
0.96 (0.34, 1.58)
29, 26.6 (21.5)
29, 26.6 (21.5)
18, 6.2 (19.7)
4.94
Leung
2014
Ctrl.
0.46 (0.31, 0.62)
0.46 (0.31, 0.62)
334, 1.61 (4.74)
334, 1.61 (4.74)
327, -.85 (5.79)
11.04
Machado
2010
Ctrl.
1.12 (0.29, 1.96)
1.12 (0.29, 1.96)
13, 1339 (664)
13, 1339 (664)
13, 740 (303)
3.34
Roelants
2004
Exerc.
-0.05 (-0.64, 0.54)
-0.05 (-0.64, 0.54)
24, 48.4 (26)
24, 48.4 (26)
20, 49.7 (21.9)
5.22
Roelants
2004
Ctrl.
0.70 (0.12, 1.28)
0.70 (0.12, 1.28)
24, 48.4 (26)
24, 48.4 (26)
25, 30.9 (23.5)
5.37
Verschueren
2004
Ctrl.
0.94 (0.35, 1.54)
0.94 (0.35, 1.54)
25, 131 (23.8)
25, 131 (23.8)
24, 111 (19)
5.22
Verschueren
2004
Exerc.
-0.05 (-0.62, 0.52)
-0.05 (-0.62, 0.52)
25, 131 (23.8)
25, 131 (23.8)
22, 133 (22.1)
5.42
von Stengel
2012
Exerc.
0.24 (-0.17, 0.65)
0.24 (-0.17, 0.65)
46, 119 (163)
46, 119 (163)
47, 84.1 (122)
7.43
von Stengel
2012
Ctrl.
0.56 (0.15, 0.97)
0.56 (0.15, 0.97)
46, 119 (163)
46, 119 (163)
48, 35.5 (132)
7.37
Subtotal (I-squared = 59.5%, p = 0.001)
0.33 (0.15, 0.51)
0.33 (0.15, 0.51)
840
840
785
100.00
with estimated predictive interval
.
. (-0.28, 0.93)
Dynamic Strength
Kennis
2013
Ctrl.
0.05 (-0.47, 0.58)
0.05 (-0.47, 0.58)
25, 86.2 (13.5)
25, 86.2 (13.5)
32, 85.3 (18.6)
11.70
Kennis
2013
Exerc.
-0.47 (-1.04, 0.09)
-0.47 (-1.04, 0.09)
25, 86.2 (13.5)
25, 86.2 (13.5)
25, 93.1 (15.1)
10.86
Mikhael
2010
Ctrl.
1.36 (0.23, 2.49)
1.36 (0.23, 2.49)
9, 142 (91.8)
9, 142 (91.8)
7, 9.59 (91.6)
4.18
Raimundo
2009
Exerc.
0.60 (-0.18, 1.37)
0.60 (-0.18, 1.37)
14, .16 (.32)
14, .16 (.32)
13, 0 (.174)
7.37
Rees
2007
Ctrl.
0.38 (-0.34, 1.11)
0.38 (-0.34, 1.11)
15, 178 (34.4)
15, 178 (34.4)
15, 163 (38.3)
8.07
Rees
2008
Exerc.
-0.03 (-0.77, 0.71)
-0.03 (-0.77, 0.71)
15, 175 (33.2)
15, 175 (33.2)
13, 176 (45.5)
7.79
Rees
2007
Exerc.
-0.04 (-0.79, 0.70)
-0.04 (-0.79, 0.70)
15, 178 (34.4)
15, 178 (34.4)
13, 180 (41.8)
7.79
Roelants
2004
Ctrl.
0.49 (-0.08, 1.06)
0.49 (-0.08, 1.06)
24, 15.6 (18.4)
24, 15.6 (18.4)
25, 6.69 (17.7)
10.73
Roelants
2004
Exerc.
-0.13 (-0.72, 0.47)
-0.13 (-0.72, 0.47)
24, 15.6 (18.4)
24, 15.6 (18.4)
20, 17.9 (17.7)
10.24
Verschueren
2004
Exerc.
-0.15 (-0.72, 0.43)
-0.15 (-0.72, 0.43)
25, 94.8 (23.8)
25, 94.8 (23.8)
22, 97.9 (16.7)
10.63
Verschueren
2004
Ctrl.
0.57 (0.00, 1.15)
0.57 (0.00, 1.15)
25, 94.8 (16.2)
25, 94.8 (16.2)
24, 85.4 (16.1)
10.66
Subtotal (I-squared = 40.2%, p = 0.080)
0.17 (-0.09, 0.42)
0.17 (-0.09, 0.42)
216
216
209
100.00
with estimated predictive interval
.
. (-0.51, 0.84)
Power
Machado
2010
Ctrl.
-0.17 (-0.94, 0.60)
-0.17 (-0.94, 0.60)
13, 94.2 (32.1)
13, 94.2 (32.1)
13, 102 (54.4)
16.21
Mikhael
2010
Ctrl.
-0.91 (-1.96, 0.15)
-0.91 (-1.96, 0.15)
9, 65.4 (54.8)
9, 65.4 (54.8)
7, 119 (57.8)
10.96
Raimundo
2009
Exerc.
0.67 (-0.11, 1.45)
0.67 (-0.11, 1.45)
14, 4.3 (12.3)
14, 4.3 (12.3)
13, -3.1 (8.61)
16.00
Rees
2008
Exerc.
0.59 (-0.17, 1.35)
0.59 (-0.17, 1.35)
15, 38.3 (7.2)
15, 38.3 (7.2)
13, 33.1 (10)
16.43
Russo
2003
Ctrl.
0.24 (-0.49, 0.97)
0.24 (-0.49, 0.97)
14, 187 (35.5)
14, 187 (35.5)
15, 179 (30.2)
17.15
Stolzenberg
2013
Ctrl.
0.62 (0.10, 1.14)
0.62 (0.10, 1.14)
30, 28.5 (5)
30, 28.5 (5)
30, 25.8 (3.4)
23.25
Subtotal (I-squared = 46.8%, p = 0.094)
0.26 (-0.15, 0.68)
0.26 (-0.15, 0.68)
95
95
91
100.00
with estimated predictive interval
.
. (-0.88, 1.41)
Rate of Force Development
Bogaerts
2007
Ctrl.
0.42 (-0.11, 0.95)
0.42 (-0.11, 0.95)
25, 18.3 (3.42)
25, 18.3 (3.42)
32, 16.5 (4.7)
9.59
Bogaerts
2007
Exerc.
-0.15 (-0.70, 0.41)
-0.15 (-0.70, 0.41)
25, 18.3 (3.42)
25, 18.3 (3.42)
25, 18.9 (5.04)
8.71
Kennis
2013
Ctrl.
0.16 (-0.37, 0.68)
0.16 (-0.37, 0.68)
25, 19.2 (4.1)
25, 19.2 (4.1)
32, 18.3 (5.77)
9.78
Kennis
2013
Exerc.
0.06 (-0.50, 0.61)
0.06 (-0.50, 0.61)
25, 19.2 (4.1)
25, 19.2 (4.1)
25, 18.9 (4.6)
8.74
Klarner
2012
Ctrl.
0.38 (-0.19, 0.96)
0.38 (-0.19, 0.96)
34, 2.8 (14.8)
34, 2.8 (14.8)
18, -2.6 (11.9)
8.08
Klarner
2012
Ctrl.
0.45 (-0.14, 1.05)
0.45 (-0.14, 1.05)
29, 3.3 (13.4)
29, 3.3 (13.4)
18, -2.6 (11.9)
7.57
Roelants
2004
Ctrl.
0.66 (0.08, 1.23)
0.66 (0.08, 1.23)
24, 41 (37.1)
24, 41 (37.1)
25, 15.3 (39.9)
8.09
Roelants
2004
Exerc.
-0.11 (-0.71, 0.48)
-0.11 (-0.71, 0.48)
24, 41 (37.1)
24, 41 (37.1)
20, 45.1 (34.8)
7.61
von Stengel
2012
Ctrl.
0.56 (0.14, 0.97)
0.56 (0.14, 0.97)
46, 1.7 (2.6)
46, 1.7 (2.6)
48, .4 (2)
15.78
von Stengel
2012
Exerc.
0.32 (-0.09, 0.73)
0.32 (-0.09, 0.73)
46, 1.7 (2.6)
46, 1.7 (2.6)
47, .8 (3)
16.04
Subtotal (I-squared = 0.0%, p = 0.454)
0.30 (0.13, 0.46)
0.30 (0.13, 0.46)
303
303
290
100.00
with estimated predictive interval
.
. (0.10, 0.49)
Functional Strength
Amaral
2014
Ctrl.
0.32 (-0.61, 1.25)
0.32 (-0.61, 1.25)
9, 16 (3)
9, 16 (3)
9, 15 (3)
5.58
Beck
2010
Ctrl.
-0.07 (-0.95, 0.81)
-0.07 (-0.95, 0.81)
17, -13.4 (4.12)
17, -13.4 (4.12)
7, -13.1 (3.87)
5.93
Beck
2010
Ctrl.
0.07 (-0.77, 0.91)
0.07 (-0.77, 0.91)
17, -12.8 (4.2)
17, -12.8 (4.2)
8, -13.1 (3.87)
6.21
Furness
2010
Ctrl.
0.96 (0.28, 1.65)
0.96 (0.28, 1.65)
19, -11.9 (2)
19, -11.9 (2)
18, -13.5 (1.1)
7.48
Furness
2009
Ctrl.
0.61 (-0.05, 1.27)
0.61 (-0.05, 1.27)
19, -12.4 (1.95)
19, -12.4 (1.95)
18, -13.4 (1.12)
7.69
Gomez-Cabello
2013
Ctrl.
0.31 (-0.25, 0.88)
0.31 (-0.25, 0.88)
24, 16.5 (3.6)
24, 16.5 (3.6)
25, 15.5 (2.6)
8.59
Iwamoto
2012
Ctrl.
0.46 (-0.13, 1.05)
0.46 (-0.13, 1.05)
26, 17.1 (21.2)
26, 17.1 (21.2)
20, 7.5 (19.3)
8.33
Lachance
2012
Exerc.
-0.52 (-1.06, 0.02)
-0.52 (-1.06, 0.02)
26, 13.5 (4.6)
26, 13.5 (4.6)
29, 16.6 (6.8)
8.83
Mikhael
2010
Ctrl.
0.27 (-0.72, 1.27)
0.27 (-0.72, 1.27)
9, -.934 (1.07)
9, -.934 (1.07)
7, -1.29 (1.41)
5.18
Raimundo
2009
Exerc.
-1.28 (-2.13, -0.44)
-1.28 (-2.13, -0.44)
14, .27 (.797)
14, .27 (.797)
13, 1.23 (.637)
6.21
Rees
2007
Ctrl.
0.99 (0.23, 1.75)
0.99 (0.23, 1.75)
15, -8.69 (.79)
15, -8.69 (.79)
15, -9.73 (1.21)
6.81
Rees
2007
Exerc.
0.26 (-0.48, 1.01)
0.26 (-0.48, 1.01)
15, -8.69 (.79)
15, -8.69 (.79)
13, -8.93 (.99)
6.95
Russo
2003
Ctrl.
-0.21 (-0.94, 0.52)
-0.21 (-0.94, 0.52)
14, 172 (19.8)
14, 172 (19.8)
15, 176 (15.9)
7.09
Stolzenberg
2013
Ctrl.
0.20 (-0.30, 0.71)
0.20 (-0.30, 0.71)
30, -1.22 (.3)
30, -1.22 (.3)
30, -1.28 (.28)
9.13
Subtotal (I-squared = 59.2%, p = 0.003)
0.18 (-0.12, 0.48)
0.18 (-0.12, 0.48)
254
254
227
100.00
with estimated predictive interval
.
. (-0.81, 1.17)
.
NOTE: Weights are from random effects analysis
-1
-.75
-.5
-.25
0
0
.25
.5
.75
1
1.5
2
Favors Control
Favors WBV
