# Supplementary material for: Effects of whole-body vibration on proxies of muscle strength in old adults: a systematic review and meta-analysis on the role of physical capacity level
Source: Eur Rev Aging Phys Act. 2015 Dec 8;12:12. doi: 10.1186/s11556-015-0158-3 (PMC4748331; doi:10.1186/s11556-015-0158-3)
Supplement: Additional file 3: — Forest plot overview of Classification Slow-Go, outcome: all strength outcomes (IMVC, DS, Power, RFD, FS). (PPTX 105 kb) [file 11556_2015_158_MOESM3_ESM.pptx]

## Slide 1
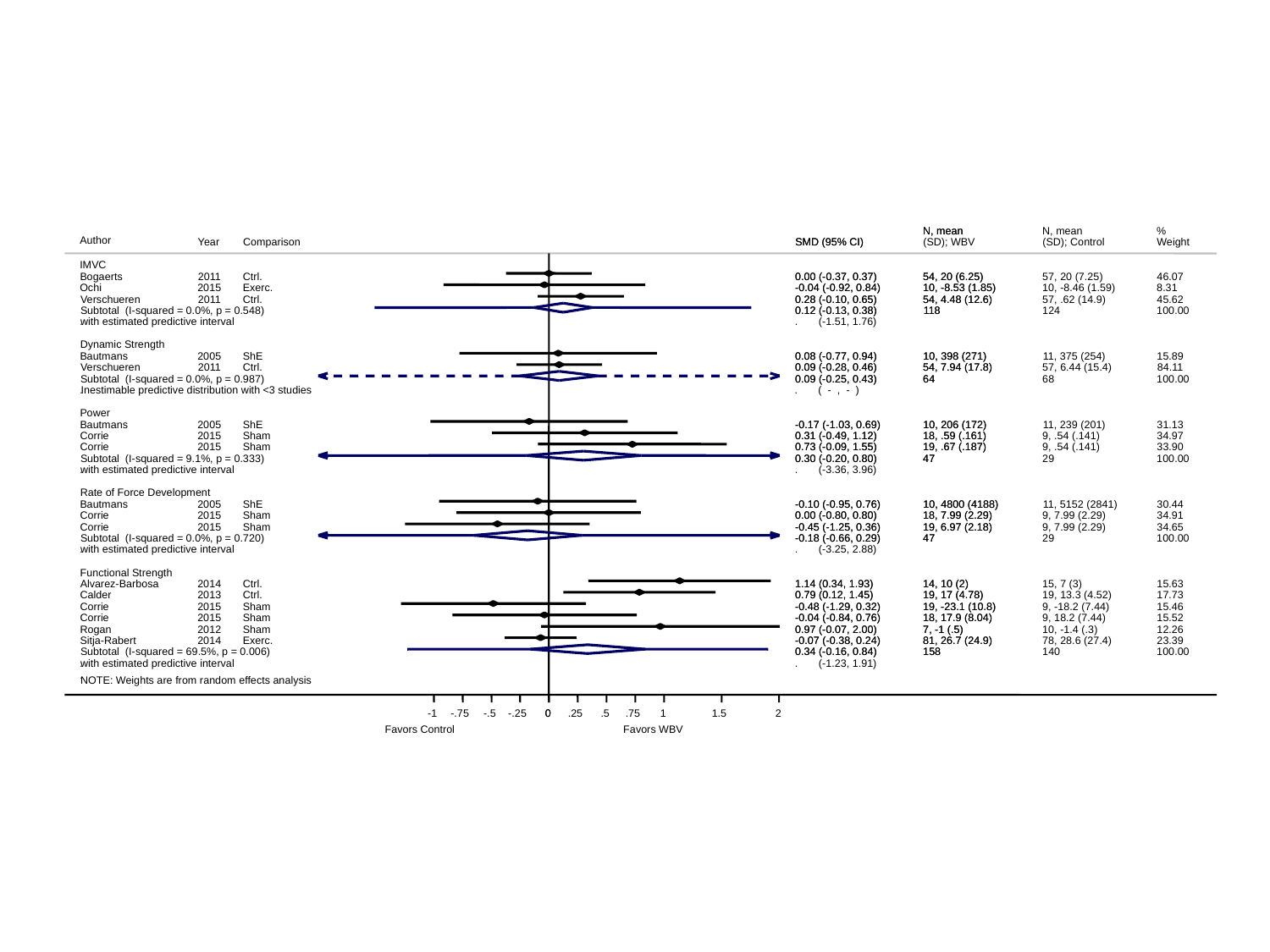

N, mean
N, mean
N, mean
%
Author
Year
Comparison
SMD (95% CI)
SMD (95% CI)
(SD); WBV
(SD); Control
Weight
IMVC
Bogaerts
2011
Ctrl.
0.00 (-0.37, 0.37)
0.00 (-0.37, 0.37)
54, 20 (6.25)
54, 20 (6.25)
57, 20 (7.25)
46.07
Ochi
2015
Exerc.
-0.04 (-0.92, 0.84)
-0.04 (-0.92, 0.84)
10, -8.53 (1.85)
10, -8.53 (1.85)
10, -8.46 (1.59)
8.31
Verschueren
2011
Ctrl.
0.28 (-0.10, 0.65)
0.28 (-0.10, 0.65)
54, 4.48 (12.6)
54, 4.48 (12.6)
57, .62 (14.9)
45.62
Subtotal (I-squared = 0.0%, p = 0.548)
0.12 (-0.13, 0.38)
0.12 (-0.13, 0.38)
118
118
124
100.00
with estimated predictive interval
.
. (-1.51, 1.76)
Dynamic Strength
Bautmans
2005
ShE
0.08 (-0.77, 0.94)
0.08 (-0.77, 0.94)
10, 398 (271)
10, 398 (271)
11, 375 (254)
15.89
Verschueren
2011
Ctrl.
0.09 (-0.28, 0.46)
0.09 (-0.28, 0.46)
54, 7.94 (17.8)
54, 7.94 (17.8)
57, 6.44 (15.4)
84.11
Subtotal (I-squared = 0.0%, p = 0.987)
0.09 (-0.25, 0.43)
0.09 (-0.25, 0.43)
64
64
68
100.00
Inestimable predictive distribution with <3 studies
.
. ( - , - )
Power
Bautmans
2005
ShE
-0.17 (-1.03, 0.69)
-0.17 (-1.03, 0.69)
10, 206 (172)
10, 206 (172)
11, 239 (201)
31.13
Corrie
2015
Sham
0.31 (-0.49, 1.12)
0.31 (-0.49, 1.12)
18, .59 (.161)
18, .59 (.161)
9, .54 (.141)
34.97
Corrie
2015
Sham
0.73 (-0.09, 1.55)
0.73 (-0.09, 1.55)
19, .67 (.187)
19, .67 (.187)
9, .54 (.141)
33.90
Subtotal (I-squared = 9.1%, p = 0.333)
0.30 (-0.20, 0.80)
0.30 (-0.20, 0.80)
47
47
29
100.00
with estimated predictive interval
.
. (-3.36, 3.96)
Rate of Force Development
Bautmans
2005
ShE
-0.10 (-0.95, 0.76)
-0.10 (-0.95, 0.76)
10, 4800 (4188)
10, 4800 (4188)
11, 5152 (2841)
30.44
Corrie
2015
Sham
0.00 (-0.80, 0.80)
0.00 (-0.80, 0.80)
18, 7.99 (2.29)
18, 7.99 (2.29)
9, 7.99 (2.29)
34.91
Corrie
2015
Sham
-0.45 (-1.25, 0.36)
-0.45 (-1.25, 0.36)
19, 6.97 (2.18)
19, 6.97 (2.18)
9, 7.99 (2.29)
34.65
Subtotal (I-squared = 0.0%, p = 0.720)
-0.18 (-0.66, 0.29)
-0.18 (-0.66, 0.29)
47
47
29
100.00
with estimated predictive interval
.
. (-3.25, 2.88)
Functional Strength
Alvarez-Barbosa
2014
Ctrl.
1.14 (0.34, 1.93)
1.14 (0.34, 1.93)
14, 10 (2)
14, 10 (2)
15, 7 (3)
15.63
Calder
2013
Ctrl.
0.79 (0.12, 1.45)
0.79 (0.12, 1.45)
19, 17 (4.78)
19, 17 (4.78)
19, 13.3 (4.52)
17.73
Corrie
2015
Sham
-0.48 (-1.29, 0.32)
-0.48 (-1.29, 0.32)
19, -23.1 (10.8)
19, -23.1 (10.8)
9, -18.2 (7.44)
15.46
Corrie
2015
Sham
-0.04 (-0.84, 0.76)
-0.04 (-0.84, 0.76)
18, 17.9 (8.04)
18, 17.9 (8.04)
9, 18.2 (7.44)
15.52
Rogan
2012
Sham
0.97 (-0.07, 2.00)
0.97 (-0.07, 2.00)
7, -1 (.5)
7, -1 (.5)
10, -1.4 (.3)
12.26
Sitja-Rabert
2014
Exerc.
-0.07 (-0.38, 0.24)
-0.07 (-0.38, 0.24)
81, 26.7 (24.9)
81, 26.7 (24.9)
78, 28.6 (27.4)
23.39
Subtotal (I-squared = 69.5%, p = 0.006)
0.34 (-0.16, 0.84)
0.34 (-0.16, 0.84)
158
158
140
100.00
with estimated predictive interval
.
. (-1.23, 1.91)
.
NOTE: Weights are from random effects analysis
-1
-.75
-.5
-.25
0
0
.25
.5
.75
1
1.5
2
Favors Control
Favors WBV
