# Supplementary material for: Effects of whole-body vibration on proxies of muscle strength in old adults: a systematic review and meta-analysis on the role of physical capacity level
Source: Eur Rev Aging Phys Act. 2015 Dec 8;12:12. doi: 10.1186/s11556-015-0158-3 (PMC4748331; doi:10.1186/s11556-015-0158-3)
Supplement: Additional file 4: — Forest plot overview of Classification No-Go, outcome: all strength outcomes (IMVC, DS, Power, RFD, FS). (PPTX 75 kb) [file 11556_2015_158_MOESM4_ESM.pptx]

## Slide 1
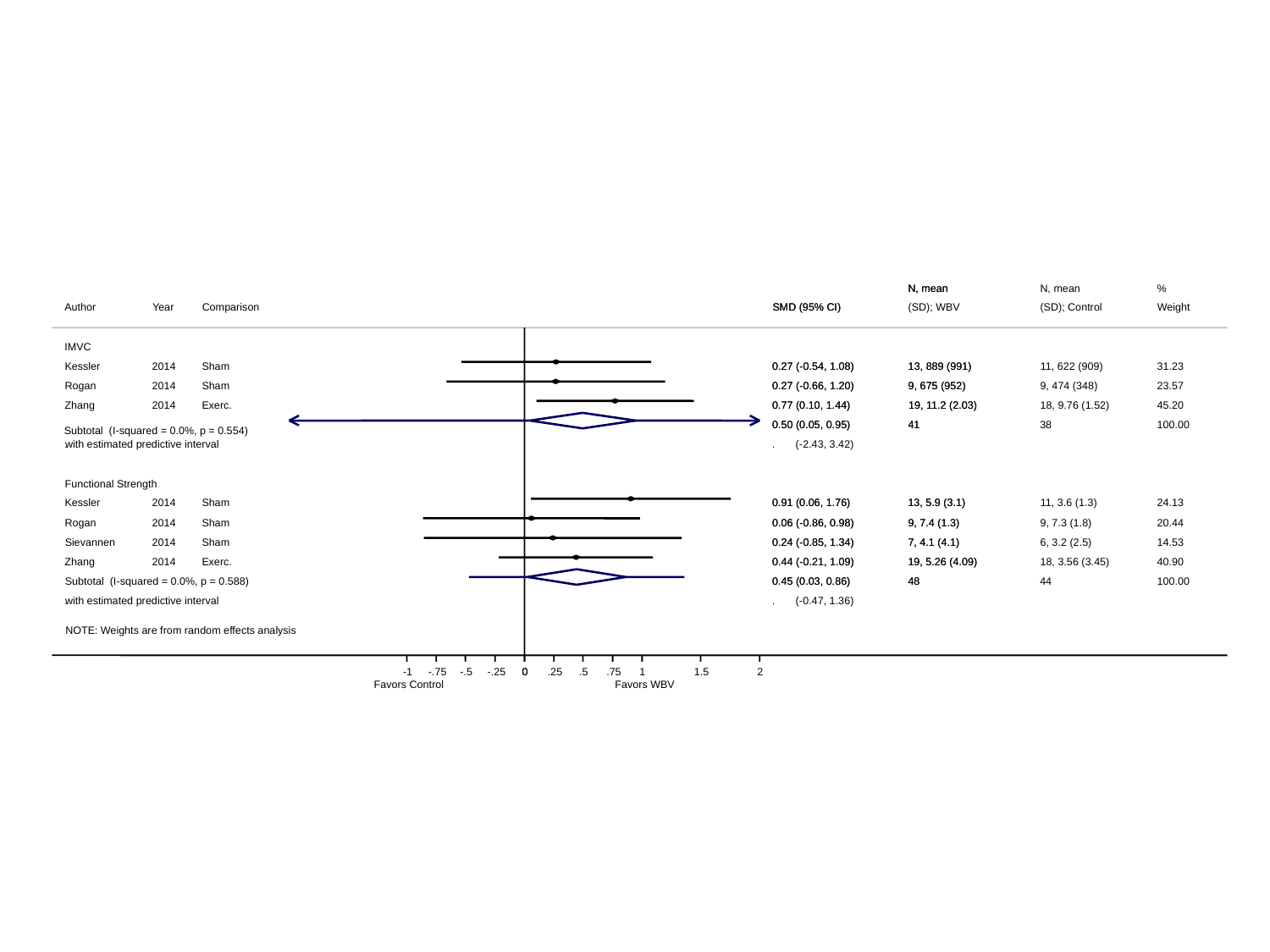

N, mean
N, mean
N, mean
%
Author
Year
Comparison
SMD (95% CI)
SMD (95% CI)
(SD); WBV
(SD); Control
Weight
IMVC
Kessler
2014
Sham
0.27 (-0.54, 1.08)
0.27 (-0.54, 1.08)
13, 889 (991)
13, 889 (991)
11, 622 (909)
31.23
Rogan
2014
Sham
0.27 (-0.66, 1.20)
0.27 (-0.66, 1.20)
9, 675 (952)
9, 675 (952)
9, 474 (348)
23.57
Zhang
2014
Exerc.
0.77 (0.10, 1.44)
0.77 (0.10, 1.44)
19, 11.2 (2.03)
19, 11.2 (2.03)
18, 9.76 (1.52)
45.20
0.50 (0.05, 0.95)
0.50 (0.05, 0.95)
41
41
38
100.00
Subtotal (I-squared = 0.0%, p = 0.554)
.
with estimated predictive interval
. (-2.43, 3.42)
Functional Strength
Kessler
2014
Sham
0.91 (0.06, 1.76)
0.91 (0.06, 1.76)
13, 5.9 (3.1)
13, 5.9 (3.1)
11, 3.6 (1.3)
24.13
Rogan
2014
Sham
0.06 (-0.86, 0.98)
0.06 (-0.86, 0.98)
9, 7.4 (1.3)
9, 7.4 (1.3)
9, 7.3 (1.8)
20.44
Sievannen
2014
Sham
0.24 (-0.85, 1.34)
0.24 (-0.85, 1.34)
7, 4.1 (4.1)
7, 4.1 (4.1)
6, 3.2 (2.5)
14.53
Zhang
2014
Exerc.
0.44 (-0.21, 1.09)
0.44 (-0.21, 1.09)
19, 5.26 (4.09)
19, 5.26 (4.09)
18, 3.56 (3.45)
40.90
Subtotal (I-squared = 0.0%, p = 0.588)
0.45 (0.03, 0.86)
0.45 (0.03, 0.86)
48
48
44
100.00
.
with estimated predictive interval
. (-0.47, 1.36)
NOTE: Weights are from random effects analysis
-1
-.75
-.5
-.25
0
0
.25
.5
.75
1
1.5
2
Favors Control
Favors WBV
